# Seminar in Epileptology: Normal awake and sleep patterns, interictal abnormalities, and ictal patterns on scalp EEG

**DOI:** 10.1002/epd2.70071

**Published:** 2025-08-09

**Authors:** Juan Luis Alcala‐Zermeno, Roohi Katyal, Birgit Frauscher, Donald Schomer, Michael R. Sperling, Roy Strowd, William O. Tatum, Elaine Wirrell, Sándor Beniczky, Fábio A. Nascimento

**Affiliations:** ^1^ Comprehensive Epilepsy Center, Department of Neurology Columbia University Irving Medical Center, New York Presbyterian Hospital New York New York USA; ^2^ Department of Neurology Louisiana State University Health Shreveport Shreveport Louisiana USA; ^3^ Department of Neurology Duke University Medical Center Durham North Carolina USA; ^4^ Department of Biomedical Engineering Duke Pratt School of Engineering Durham North Carolina USA; ^5^ Department of Neurology Beth Israel Deaconess Medical Center, Harvard Medical School Boston Massachusetts USA; ^6^ Jefferson Comprehensive Epilepsy Center, Department of Neurology Thomas Jefferson University Philadelphia Pennsylvania USA; ^7^ Department of Neurology Wake Forest University School of Medicine Winston‐Salem North Carolina USA; ^8^ Department of Neurology Mayo Clinic Jacksonville Florida USA; ^9^ Divisions of Child and Adolescent Neurology and Epilepsy, Department of Neurology Mayo Clinic Rochester Minnesota USA; ^10^ Department of Clinical Neurophysiology Aarhus University Hospital Aarhus Denmark; ^11^ Department of Clinical Medicine Aarhus University Aarhus Denmark; ^12^ Department of Clinical Neurophysiology Danish Epilepsy Centre Dianalund Denmark; ^13^ Department of Neurology Washington University School of Medicine St. Louis Missouri USA

**Keywords:** benign, dipole, epilepsy, electroencephalogram, epileptiform, seizure

## Abstract

The accurate interpretation of scalp EEG remains an instrumental diagnostic component of epilepsy care. Knowledge of what constitutes normal EEG findings, non‐epileptiform abnormalities, and epileptiform patterns—both ictal and interictal—is essential for appropriate patient management. The International League Against Epilepsy (ILAE) has developed an educational curriculum containing learning objectives necessary to accurately diagnose and manage people with epilepsy. In this Seminar in Epileptology, we address the learning objectives related to assessment and interpretation of EEG background activity, normal sleep patterns, provocation methods, interictal abnormalities, and ictal patterns, as well as a brief discussion on relevant advances in scalp EEG analysis and future perspectives on this topic.

**Key points**

- The posterior dominant rhythm is a well‐formed sinusoidal pattern seen in the occipital leads of an awake EEG. It appears when the eyes are closed and during relaxation, typically ranging between 8 and 12 Hz in adults.
- During drowsiness, the EEG shows a decrease in alpha activity and an increase in centrally predominant polymorphic theta activity (5–8 Hz), along with slow, horizontal, roving eye movements.
- Vertex sharp waves are central sharp transients that typically appear symmetrically and last less than 0.5 seconds. These waveforms may appear in isolation or in runs at 2–6 Hz.
- The presence of generalized polymorphic delta (0.5–4 Hz) slowing outside of slow‐wave sleep, and especially during wakefulness, is considered abnormal in an adult EEG and is a hallmark of diffuse cerebral dysfunction.
- The six IFCN criteria for identifying interictal epileptiform discharges include sharp or spiky wave morphology, different wave duration than ongoing background, waveform asymmetry, an associated aftergoing slow wave, disruption of background activity, and a distribution suggesting a brain source.
- Hypsarrhythmia is the hallmark interictal EEG pattern of infantile epileptic spasms syndrome, characterized by diffuse, high‐amplitude, disorganized slow waves with multifocal epileptiform discharges.
- An electrographic seizure is defined by the ACNS as (i) epileptiform discharges averaging >2.5 Hz for at least 10 seconds or (ii) any pattern with definite evolution and lasting at least 10 seconds. Evolution is characterized by the presence of at least two unequivocal, sequential changes in at least one domain: a frequency change of at least 0.5 Hz twice and in the same direction, a morphology change to a new pattern twice, or the spread into or out of two electrode locations.
- The description of ictal rhythms for localizing temporal lobe seizures, as proposed by Ebersole and Pacia in 1995, includes three basic patterns: Type 1 (hippocampal onset; inferotemporal regular 5‐9 Hz rhyhtm), Type 2 (neocortical onset; lateralized hemispheric irregular 2‐5 Hz rhythm), and Type 3 (neocortical onset; unlateralized or diffuse irregular changes).
- Provocative maneuvers, such as hyperventilation and photic stimulation, are integral to standard EEG procedures to help diagnose epilepsy by eliciting specific responses.

## 1 INTRODUCTION

Since 1933, when Hans Berger published the first EEG recording of a patient having an absence seizure—work later expanded by Gibbs and Lennox using early EEG technology to study epilepsy—scalp EEG has remained an essential tool in the diagnosis and care of patients with seizures and epilepsy.^1^, ^2^ Besides the accurate and reliable interpretation of normal graphoelements in awake and sleep recordings,^3^ it is crucial to be able to interpret two major groups of abnormal EEG findings: abnormalities that occur between seizures (interictal) and during seizures (ictal). Inability to accurately and reliably interpret EEG findings may result in the under‐recognition of epilepsy cases, or—what is more common and problematic—in mislabeling normal findings as abnormal patterns. The latter scenario often leads to unnecessary and harmful medical interventions that may take years to undo.^4^

This Seminar in Epileptology begins with an overview of what constitutes a normal EEG during wakefulness and sleep across routine, ambulatory, and epilepsy monitoring unit (EMU) settings, followed by the most common interictal epileptiform and non‐epileptiform abnormalities. It then provides a framework to identify seizures as well as a description of the most common ictal patterns. The seminar concludes on the topic of provocation maneuvers and their clinical utility as well as future directions for electroencephalography. The goal of this seminar is to address the following International League Against Epilepsy (ILAE) learning objectives^5^: *1.4.5 Recognize the indications for the different types of provocation methods; 1.4.7 Recognize and describe background activity and sleep patterns in all age groups; 1.4.9 Recognize and describe interictal abnormalities;* and *1.4.10 Recognize and describe ictal patterns*. Although this seminar discusses findings that are pertinent to both pediatric and adult recordings, it does not address neonatal EEG findings, which have unique and age‐specific graphoelements. There are extensive reviews on neonatal EEG where this information can be found.^6^, ^7^, ^8^

## 2 NORMAL EEG IN WAKEFULNESS AND SLEEP

### 2.1 Normal awake EEG

The interpretation of scalp EEG should follow a systematic approach to ensure all relevant components of the recording are analyzed.^9^ The EEG reader begins by identifying the presence of any background activity. If absent, the interpreter must ensure that the filters are adequate and that the scalp electrodes are in place, the latter being easily performed by simply looking at the video portion of the EEG, which is widely available in most centers. Only then can we define suppression if all activity is <10 μV.^10^ If there is clear background activity present, the next step involves assessing the posterior dominant rhythm (PDR) and organization of the background.

#### 2.1.1 Posterior dominant rhythm

**FIGURE 1:**
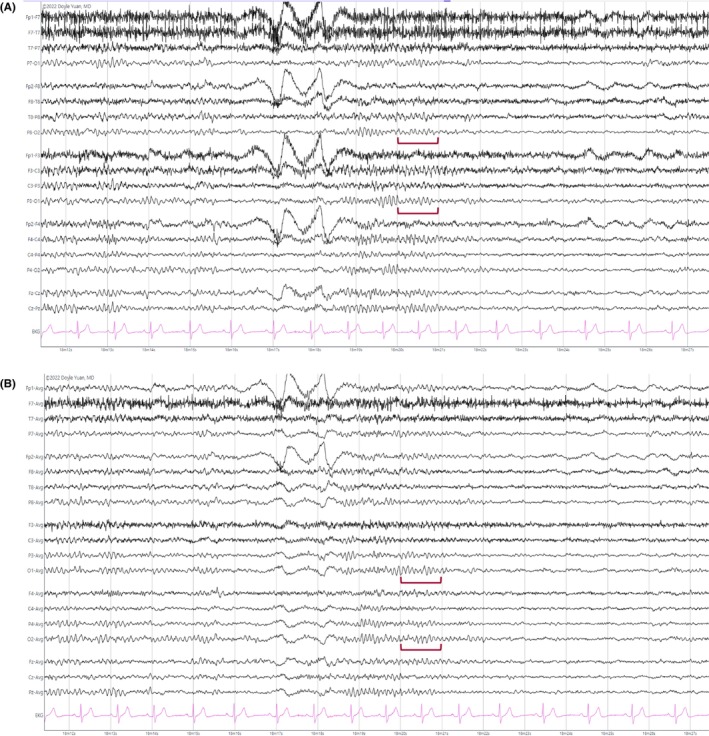
Normal awake EEG of a 32‐year‐old patient. Note the 10‐Hz posterior dominant rhythm in the 10th second of the strip (red brackets). Panel (A): bipolar longitudinal montage; panel (B): common average referential montage. Low‐frequency filter 1 Hz, high‐frequency filter 70 Hz, sensitivity 7 μV/mm for panel A and 10 μV/mm for panel B.

The hallmark, and likely the first thing to notice in an EEG during relaxed wakefulness, is the PDR, also referred to as alpha rhythm in adults (Figure 1). This is a well‐formed sinusoidal pattern observed in the posterior head region. It is most prominent in the occipital leads and is a reactive rhythm that accentuates with eye closure and attenuates with eye opening.^11^ The normal frequency varies according to age; in adults, it usually ranges between 8 and 12 Hz (Table 1).^12^ Rarely, the PDR may occur in harmonic or subharmonic frequencies of the fundamental PDR frequency. These are referred to as slow alpha variant or fast alpha variant, respectively. For instance, if the alpha frequency is 9 Hz, the first subharmonic would be 4–5 Hz and the second harmonic would be 18 Hz.^13^ The frequency of PDR should be largely synchronous and symmetric with ≤1 Hz difference between hemispheres. Amplitude, however, may vary between the left and right occipital regions. In neurotypical individuals, the frequency usually does not decrease with aging.^14^ An asymmetry in amplitude is common, with lower amplitude usually seen in the left occipital region. This asymmetry in amplitude is considered normal as long as the difference does not exceed 50% when the right has a higher amplitude and 35% when the left has a higher amplitude.^15^ However, an isolated amplitude asymmetry without a difference in frequency has not been reliably associated with pathology—unless the asymmetry is due to complete suppression on one side.

**TABLE 1:** Reference to accepted normal posterior dominant rhythm peak frequencies over age.

Age	Frequency (Hz)
≤1 year	Mean 5.3, range 3.5–7.1^a^
2–3 years	Mean 6.8, range 5–8.6
4–5 years	Mean 7.9, range 6.1–9.7
6–7 years	Mean 8.7, range 6.9–10.5
8–15 years	Mean 9.5, range 7.7–11.3
16–50 years	Mean 9.9, range 8.1–11.7
>51 years	Mean 9.1, range 7.3–10.9

*Note*: Adapted from: Lodder and van Putten.^12^

^a^ Posterior dominant rhythm (PDR) first appears in the third and fourth months of life.

#### 2.1.2 Mu rhythm

**FIGURE 2:**
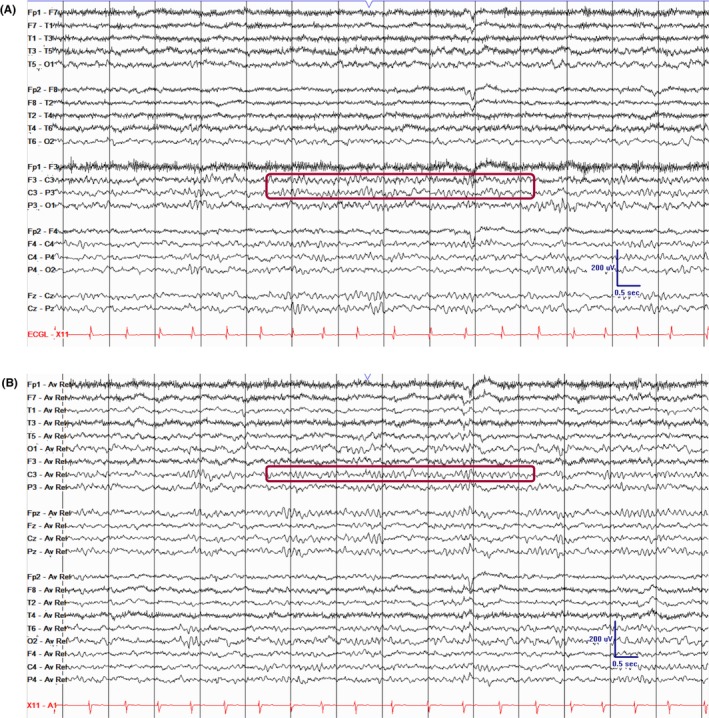
Mu rhythm on the left central region (rectangle) of a 31‐year‐old male. Panel (A): bipolar longitudinal montage; panel (B): common average referential montage. Low‐frequency filter 1 Hz, high‐frequency filter 70 Hz, sensitivity 10 μV/mm.

The mu rhythm is an arciform rhythm that occurs predominantly in the central regions and that resembles the Greek letter “μ” (Figure 2).^13^ Mu tends to occur in short runs (0.5–2 s) that are often bilateral with a tendency to shift from side to side.^16^ Mu appears predominantly during relaxed wakefulness and, less commonly, during stages 1 and 2 of non‐REM sleep. The mu rhythm may be suppressed by moving a limb on the opposite side of the body or by thinking about moving such limb. Its frequency ranges between 7 and 12 Hz, and is usually 9–10 Hz.^16^ It has been postulated that the mu rhythm represents the motor cortex like the PDR represents the occipital cortex and indeed, in source localization studies, the mu rhythm represents a dipole in the ipsilateral perirolandic cortex.^17^

#### 2.1.3 Lambda waves

**FIGURE 3:**
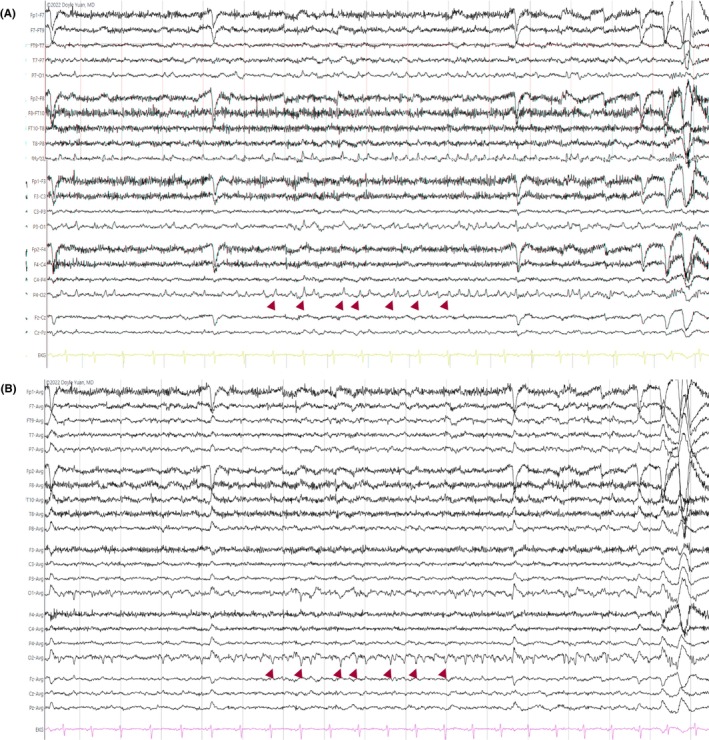
Lambda waves (arrowheads). Note the change in vertical direction on the referential montage indicating their positive polarity. Panel (A): bipolar longitudinal montage; panel (B): common average referential montage. Low‐frequency filter 1 Hz, high‐frequency filter 70 Hz, sensitivity 5 μV/mm.

Lambda waves are sharply contoured, typically surface positive, diphasic waveforms observed during wakefulness in the occipital leads that resemble the Greek letter “λ,” after which they are named.^16^, ^18^ Active visual exploration with eye movement, typically of complex images, activates lambda waves, which are bilateral, synchronous, and maximal in the occipital leads (O1, O2) either in isolation or in runs at 2–5 Hz (Figure 3). In source imaging studies, lambda waves arise from the mesial occipital cortex and are believed to represent visual evoked potentials.^17^, ^19^

#### 2.1.4 Beta

**FIGURE 4:**
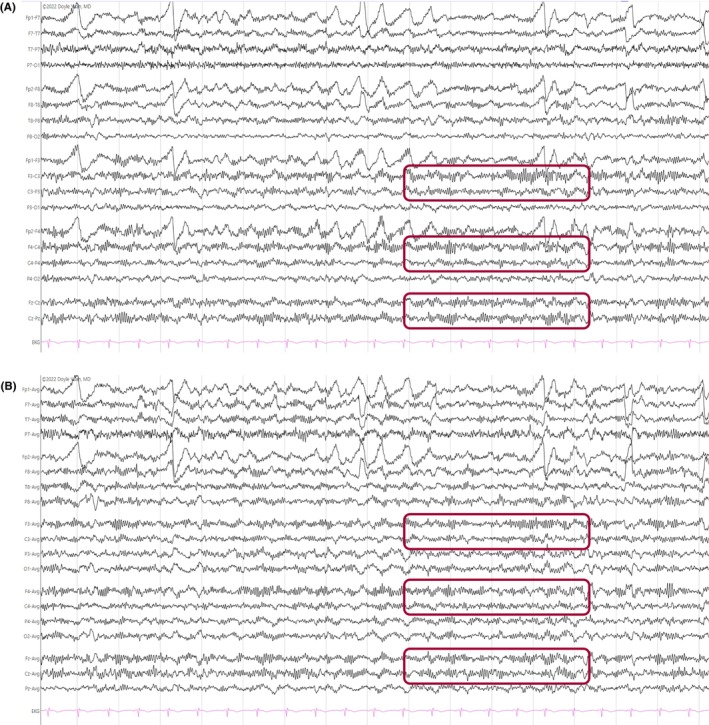
Diffuse excessive beta activity in a 29‐year‐old male with a history of polysubstance use disorder. In this example, the excess beta activity is most obvious in the frontocentral regions (red rectangles), Panel (A): bipolar longitudinal montage; panel (B): common average referential montage. Low‐frequency filter 1 Hz, high‐frequency filter 70 Hz, sensitivity 5 μV/mm.

Beta activity, a normal finding seen in healthy individuals, is characterized by low‐amplitude 13–25 Hz activity seen maximally over the frontocentral regions that becomes accentuated during periods of drowsiness and sleep. During wakefulness, beta becomes more noticeable in periods of hypervigilance or complex cognitive processing; however, it may be a normal trait of wakefulness in the EEG of some healthy individuals, regardless of their cognitive state.^18^ A marked increase in beta activity is typically provoked by sedative medications, including benzodiazepines, propofol, and barbiturates. The excess beta activity induced by medications has a widespread spatial distribution beyond the frontocentral region (Figure 4).^20^ Central nervous system stimulants, including cocaine and amphetamines, and tricyclic antidepressants, can also result in an increase in diffuse beta activity. Typically, medications tend to produce more prominent beta activity in children than in adults.^20^ Additionally, increased beta has also been reported in certain neuropsychiatric and neurodevelopmental conditions, such as attention deficit hyperkinetic disorder,^21^ schizophrenia,^22^ and Dup15q syndrome, which is strongly associated with autism spectrum disorder.^23^

### 2.2 Normal EEG in drowsiness and sleep

The transition between wakefulness and sleep represents the drowsy state, which has a wide array of pattern changes and normal variants that can be mistaken for epileptiform abnormalities.^3^, ^24^ Readers may find an in‐depth discussion on normal variants and how they may be misinterpreted as epileptiform abnormalities in the seminar by Amin et al. entitled “Normal variants and artifacts: importance in EEG interpretation.”^3^

During drowsiness and sleep, the EEG can be classified into two main phases: Non‐Rapid Eye Movement (NREM) and Rapid Eye Movement (REM) sleep, each characterized by distinct findings that help in their identification (Table 2). NREM sleep has three stages: N1 (which includes drowsiness), N2, and N3.^25^ The N1 stage makes up 5%–10% of the sleep state and usually progresses to N2, which represents 50% of the sleep recording (both N1 and N2 are also known as light sleep); N3, or slow‐wave sleep (deep sleep), makes up about 20% of the sleep recording, and the remaining 20%–25% is occupied by REM sleep.^26^ These percentages change with age, as older individuals have less deep sleep. In adults, sleep occurs in cycles, each lasting an average of 90–100 min. Recording sleep and awake‐sleep transitions during an EEG has been shown to increase the likelihood of detecting epileptiform activity.^27^, ^28^ Notably, in many cases, epileptiform discharges may be only recorded during sleep stages.^29^

**TABLE 2:** EEG findings during drowsiness and sleep.

	Drowsy state (early N1)	N1	N2	N3	REM sleep
Beta (frontocentral, in small amounts)					
Vertex waves					
POSTS					
Sleep spindles					
K‐complexes					
Polymorphic theta^a^					
Polymorphic delta, generalized					
Sawtooth waves					

*Note*: Blue = characteristic sleep stage when waveform/pattern appears. Yellow = less frequently seen during such stages, but still considered normal.

Abbreviations: NREM, Non‐Rapid Eye Movement sleep; POSTS, positive occipital sharp transients of sleep; REM, Rapid Eye Movement.

^a^ Polymorphic theta can be either frontocentral or generalized.

#### 2.2.1 Normal EEG in drowsiness

**FIGURE 5:**
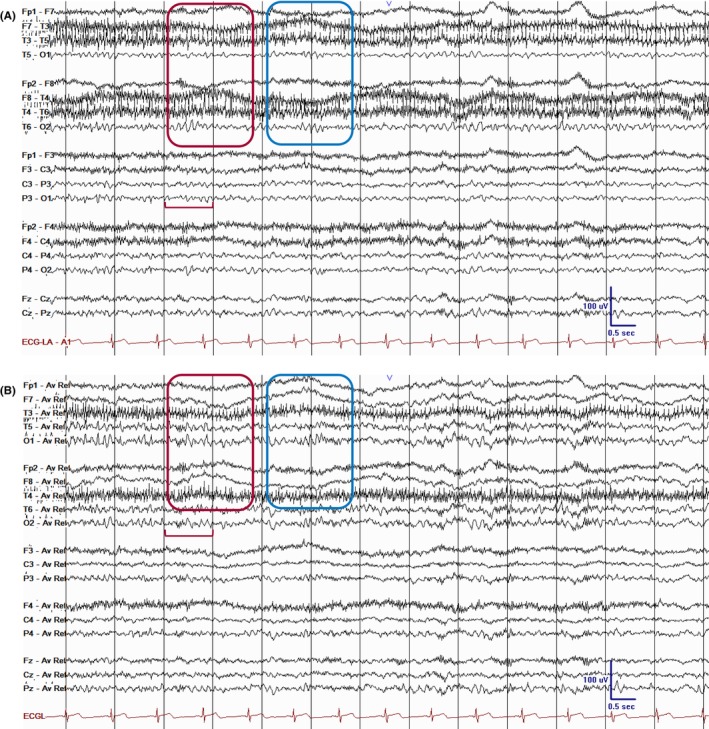
Early N1 sleep (drowsiness). Note the slow roving eye movements (red rectangle—left horizontal roving eye movement; blue rectangle—right horizontal roving eye movement) and slowing of the posterior dominant (red bracket). Panel (A): bipolar longitudinal montage; panel (B): common average referential montage. Low‐frequency filter 1 Hz, high‐frequency filter 70 Hz, sensitivity 5 μV/mm.

Transitions in states of consciousness result in changes in frequency, distribution, and amplitude of the dominant background activity. The onset of drowsiness is marked by a decrease in alpha activity, both in frequency and amplitude, an increase in centrally predominant polymorphic theta activity (5–8 Hz), which should not exceed 30% of the recording in the adult awake state^30^ and slow (0.25–0.5 Hz), pendular, horizontal, slow roving eye movements (Figure 5). Additionally, low‐amplitude frontocentral fast activity, usually in the beta frequency range (18–35 Hz), can be observed in a normal EEG during drowsiness and may progress into light sleep.^24^ Infrequently, rapid, low‐amplitude horizontal eye movements appear early in drowsiness.^24^

**FIGURE 6:**
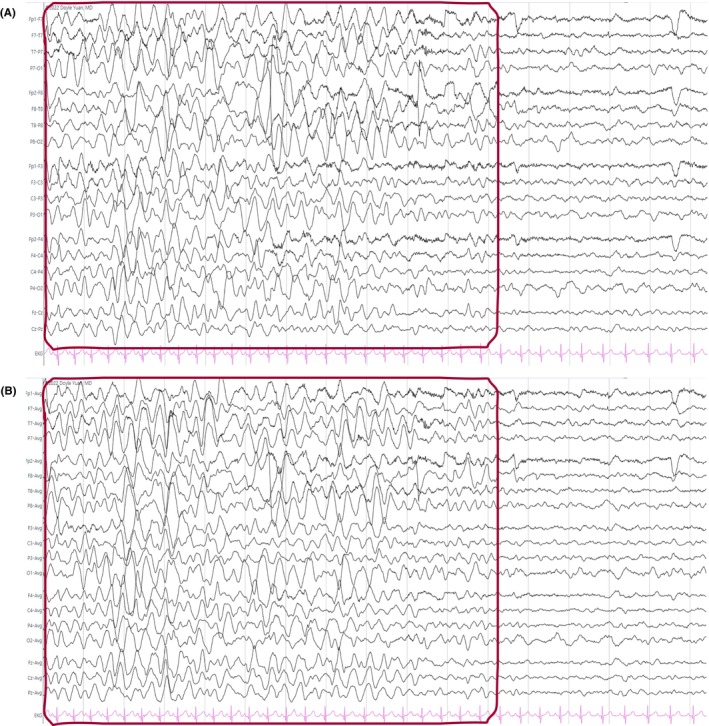
Hypnagogic hypersynchrony in a 3‐year‐old patient (red rectangle). Panel (A): bipolar longitudinal montage; panel (B): common average referential montage. Low‐frequency filter 1 Hz, high‐frequency filter 70 Hz, sensitivity 15 μV/mm.

In infancy and childhood, it is common to find high‐amplitude bursts of either frontally predominant or generalized monomorphic, delta to theta range activity in the sleep–wake transitions (Figure 6).^31^ This finding is known as hypnagogic hypersynchrony when transitioning from wakefulness to sleep, and hypnopompic hypersynchrony when transitioning from sleep to wakefulness. At times, these hypersynchronous bursts can have superimposed faster frequencies giving them a notched/spiky appearance; however, hypnic hypersynchrony should never be associated with clear spikes preceding the slow waves as these may be indicative of generalized interictal epileptiform discharges (IED).^32^ While hypnic hypersynchrony is primarily observed in children and adolescents, young adults may rarely have a *forme fruste* of this phenomenon characterized by low‐amplitude theta or delta bursts during sleep–wake transitions.^24^

#### 2.2.2 Vertex sharp waves (V‐waves)

**FIGURE 7:**
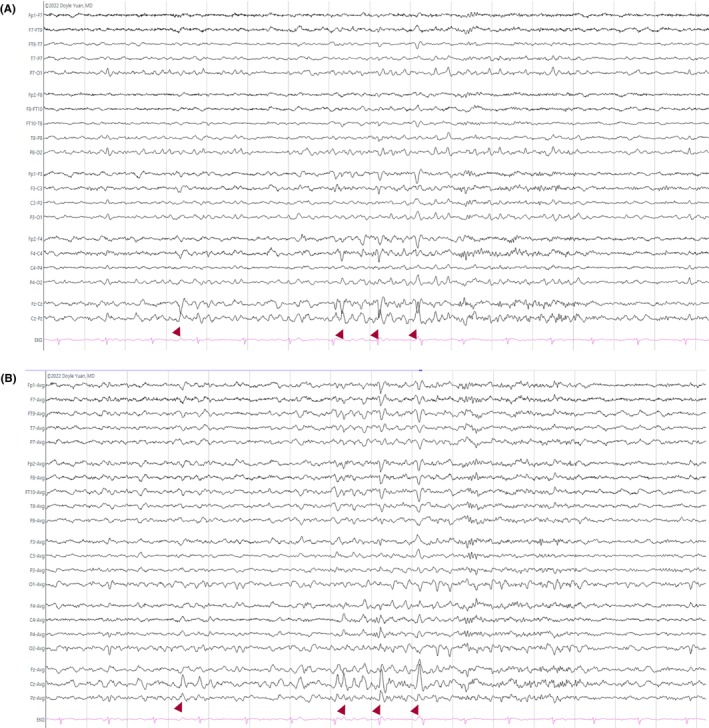
Vertex waves (arrowheads) in a 20‐year‐old patient. Panel (A): bipolar longitudinal montage; panel (B): common average referential montage. Low‐frequency filter 1 Hz, high‐frequency filter 70 Hz, sensitivity 7 μV/mm.

Vertex waves are central sharp transients that are most prominent in the midline region (Cz) and are appreciated during stages N1 and N2 (Figure 7). These waveforms start to appear between 4 and 6 months of age, and they typically exhibit a symmetric field involving the central electrodes and last <0.5 s in duration.^25^ Their morphology also changes with time, with a high‐amplitude, sharply contoured morphology transitioning into spiky transients during early infancy. They remain spiky during childhood and gradually become more blunt and lower in amplitude in adolescence and adulthood.^33^ Vertex sharp waves may appear as trains of repetitive waveforms at a frequency of 1–3 Hz, especially in children, and may have a fluctuating asymmetry.^18^

#### 2.2.3 Sleep spindles

**FIGURE 8:**
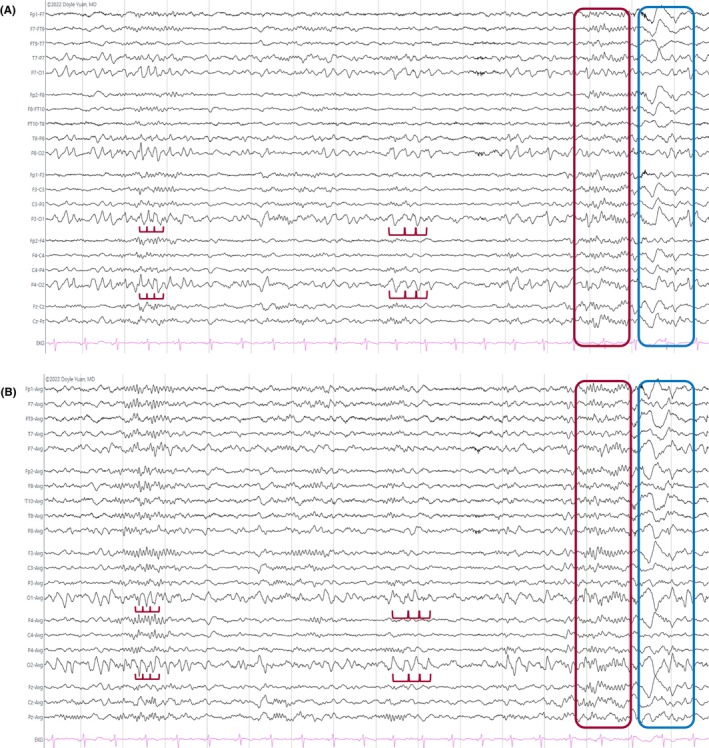
N2 sleep with different sleep graphoelements. The red brackets highlight the presence of POSTS. The red rectangle highlights a symmetric 13–14 Hz spindle. The blue rectangle highlights a K‐complex that is not associated with an arousal. Panel (A): bipolar longitudinal montage; panel (B): common average referential montage. Low‐frequency filter 1 Hz, high‐frequency filter 70 Hz, sensitivity 7 μV/mm.

Sleep spindles, a hallmark of stage N2 sleep, are 0.5–1‐second bursts of rhythmic activity over the central region.^25^ They can be classified into fast (12–15 Hz) spindles with a central maximum and slow (9–12 Hz) spindles with a more anterior distribution (Figure 8).^34^ Occasionally, they can also be seen during N3. Sleep spindles tend to have an amplitude variation giving them a sinusoidal appearance (spindle‐like). Through functional imaging experiments, sleep spindles have been identified as an integral part of sleep‐related memory encoding through rich connections between the thalamus, temporal lobe, cingulate, and paracentral cortex.^35^ Rudimentary spindles may be seen as early as in premature infants, but their morphology becomes better defined by months 3–9, when they develop the more typical comb‐like appearance. By month 6, spindles commonly have fluctuating asymmetry, and they gradually become more bisynchronous during the remainder of the first year of life. By months 12–18, spindles are expected to be bilaterally synchronous and symmetric.^36^ After 2 years of age, consistent asymmetry of sleep spindles is considered abnormal.^37^

#### 2.2.4 K‐complexes

K‐complexes are central and midline predominant, diphasic delta waves with a high‐voltage negative phase followed by a smaller positive phase, often with a superimposed sleep spindle, seen during N2 and N3 (Figure 8). However, it is only when they are not accompanied by an arousal that K‐complexes are considered truly an element of N2 sleep.^25^ Also, given their temporal relationship with arousals, K‐complexes have been associated with somatosensory activation and, as a result, they have been implicated in the preservation of sleep.^38^ In patients with generalized epilepsy and, less commonly, focal epilepsy, K‐complexes may be coupled with either generalized^39^ or focal epileptiform discharges.^40^

#### 2.2.5 Positive occipital sharp transients of sleep

Positive occipital sharp transients of sleep (POSTS) are seen during N1 and N2 and in N3.^41^ POSTS are biphasic (reversed check mark), surface positive waves seen maximally over the occipital leads that tend to be symmetric in two thirds of cases (Figure 8).^41^ These waveforms commonly appear in 0.5–2‐second trains at a frequency of 4–6 Hz.^16^ Morphologically, they are similar to lambda waves, which, as discussed above, are exclusively seen during wakefulness.^18^ POSTS have been associated with the presence of a functional visual cortex and are commonly absent in individuals with poor visual acuity bilaterally (20/200 vision or worse).^42^

#### 2.2.6 Slow‐wave sleep (N3)

**FIGURE 9:**
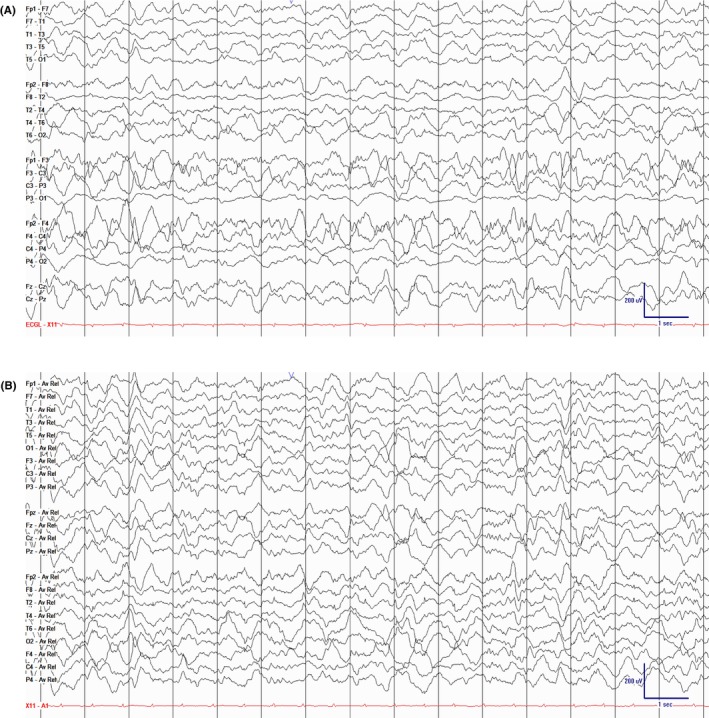
Normal N3 sleep in a 10‐year‐old patient. Note the high‐amplitude diffuse delta waveforms. Panel (A): bipolar longitudinal montage; panel (B): common average referential montage. Low‐frequency filter 1 Hz, high‐frequency filter 70 Hz, sensitivity 5 μV/mm.

High‐amplitude (≥75 μV), slow‐wave (0.5–2 Hz) activity defines N3, also known as slow‐wave sleep or deep sleep (Figure 9).^25^, ^43^ In the past, the Rechtschaffen and Kales nomenclature separated slow‐wave sleep into stages 3 and 4 based on the percentage of the epoch occupied by polymorphic high‐amplitude delta activity, but this distinction did not add much clinical value.^44^ Eventually, all slow‐wave activity was considered slow‐wave sleep as long as it filled at least 20% of a given epoch (a 30‐second strip recording on a polysomnogram).^25^ Slow‐wave sleep is one of the few instances where delta waveforms are considered normal.

#### 2.2.7 Rapid eye movement sleep

**FIGURE 10:**
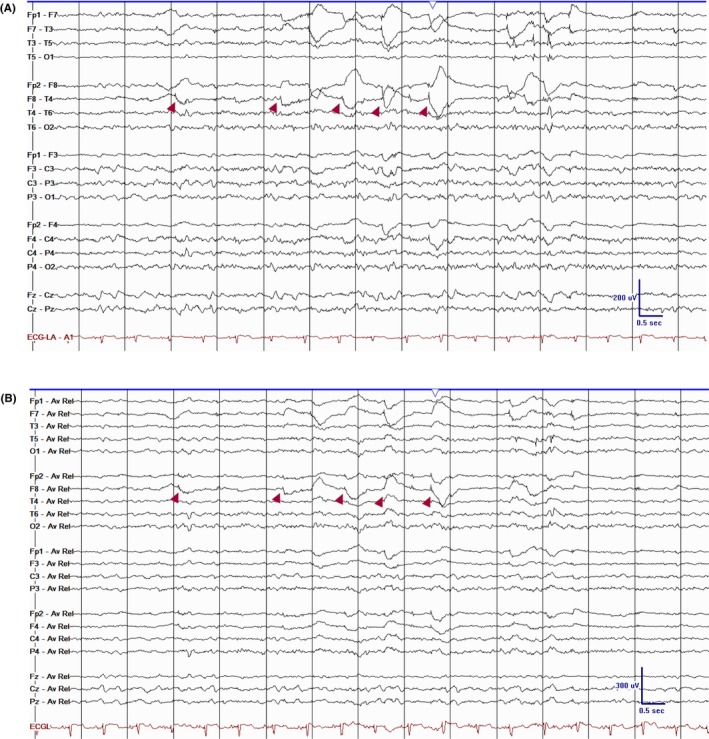
Normal Rapid Eye Movement (REM) sleep in a 42‐year‐old female. The arrowheads highlight rapid eye movements preceded by lateral rectus spikes. Panel (A): bipolar longitudinal montage; panel (B): common average referential montage. Low‐frequency filter 1 Hz, high‐frequency filter 70 Hz, sensitivity 10 μV/mm for panel A, 15 μV/mm for panel B.

REM sleep is characterized by a background of low‐amplitude mixed frequency polymorphic waveforms, usually in the theta to alpha frequency range, and a characteristic paucity of myogenic artifact resultant from physiologic atonia.^25^ A key element of REM sleep is the presence of rapid saccadic eye movement artifacts in the frontal leads that may be associated with lateral rectus spikes. The latter are low‐amplitude spiky waveforms that are artifactual and represent motor unit action potentials in the lateral rectus muscle. Given that REM is normally accompanied by atonia, lateral rectus spikes are mostly evident during this sleep stage.^18^ Sawtooth waves are asymmetric monomorphic waveforms at the vertex region with a steep upslope and a gradual downslope that give them a serrated appearance. These waveforms occur in runs at 2–6 Hz frequency (Figure 10). Sawtooth waves at the vertex are seen in up to 60% of patients during REM sleep.^45^

In addition, there are several normal variants that occur during drowsiness and sleep and tend to be misinterpreted as pathologic findings such as, but not limited to, wicket waves (misidentified as focal epileptiform discharges in the temporal region), 14 & 6 Hz positive bursts (misidentified as generalized epileptiform discharges), and Ciganek rhythm (misidentified as midline seizures). These are thoroughly covered on a different seminar entitled “Normal variants and artifacts: importance in EEG interpretation.”^3^

## 3 INTERICTAL FINDINGS

Most routine EEGs performed in patients with epilepsy will not capture a seizure given the brief duration of recording^29^ and performing long‐term studies is generally not cost‐effective at the current stage of technology. Therefore, the interpretation of abnormalities that may arise between seizures (interictal) becomes critical for epilepsy care. This section will initially focus on the most important interictal patterns, namely IEDs. Then, we will discuss abnormal interictal slowing, special patterns, and rhythmic and periodic patterns in critically ill patients.

### 3.1 Interictal epileptiform discharges

#### 3.1.1 Definition and clinical implications

Interictal epileptiform discharges are abnormal, transient, morphologically distinct waveforms that can be distinguished from ongoing background activity (Table 3).^46^ Although IEDs have a high specificity for epilepsy, their morphology and prevalence can be heterogeneous and thus open to interpretation (or misinterpretation). Identification of IEDs can help classify the epilepsy type^47^, ^48^ and aid in the identification of the epilepsy syndrome.^48^, ^49^ Regarding the latter, Table 4 shows a curated summary of the ILAE‐recognized epilepsy syndromes with mandatory diagnostic EEG criteria as per the latest guidelines, excluding neonatal onset syndromes. Note that there are additional syndromes not included in the table as they do not have mandatory diagnostic EEG criteria.^50^, ^51^, ^52^, ^53^, ^54^ Identification of IEDs can also inform the management of antiseizure medications (ASMs), including guidance on ASM withdrawal in seizure‐free patients.^47^, ^55^, ^56^ Furthermore, the presence of IEDs following a new‐onset seizure is diagnostically important as it indicates a high risk of seizure recurrence (>60% over the following 10 years).^57^ Lastly, an interesting observation from in vitro studies is that IEDs may also act to help inhibit seizures.^58^, ^59^ However, this remains to be proven in clinical practice.

**TABLE 3:** Summary of interictal epileptiform discharges.

IED	Schematic	EEG definition	Commonly seen in
Spike	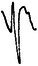	Pointed peak seen with conventional time scaling lasting 20–70 msec as per IFCN criteria	Focal epilepsies, multifocal epilepsies, generalized epilepsies
Polyspikes	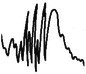	Sequence of two or more consecutive spikes lasting <0.5 s	Focal epilepsies, multifocal epilepsies, generalized epilepsies
Sharp wave	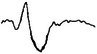	Pointed peak seen with conventional time scaling lasting 70–200 msec as per IFCN criteria	Focal epilepsies, multifocal epilepsies, generalized epilepsies
Spike‐wave complex or	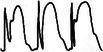	Spike, sharp wave or polyspike immediately followed by a slow wave	3 Hz (range 2–4 Hz) generalized spike–wave discharges—CAE
Sharp‐and‐wave complex or	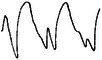		≤2.5 Hz generalized spike–wave discharges—LGS
Polyspike‐wave‐complex	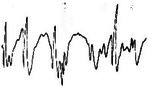		3–5.5 Hz generalized spike/polyspike–wave discharges—JME and GTCA 2–6 Hz generalized spike/polyspike–wave discharges—EMAtS
Paroxysmal fast activity	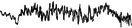	Burst of low‐amplitude spikes, occurring focally or more broadly, at frequencies of 10–25 Hz, lasting 2–10 s	Focal epilepsies, generalized epilepsies, multifocal epilepsies and DEEs, such as LGS
Hypsarrhythmia^a^	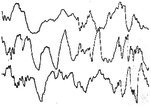	Diffuse, high‐amplitude (>200 μV), disorganized slow waves with multifocal epileptiform discharges	Infant epileptic spasm syndrome

Abbreviations: CAE, childhood absence epilepsy; DEEs, developmental and/or epileptic encephalopathies; EMAtS, Epilepsy with myoclonic‐atonic seizures; GTC, generalized tonic–clonic seizures; GTCA, epilepsy with generalized tonic–clonic seizures alone; IED, interictal epileptiform discharge; IFCN, International Federation of Clinical Neurophysiology; JME, juvenile myoclonic epilepsy; LGS, Lennox–Gastaut syndrome.

^a^ Represents a pattern rather than simple graphoelements.

**TABLE 4:** Epilepsy syndromes recognized by the International League Against Epilepsy (ILAE) with mandatory EEG criteria required for their diagnosis.

Epilepsy syndrome	Mandatory EEG criteria	Alert EEG findings^a^	Exclusionary EEG findings	Age of onset
Idiopathic generalized epilepsy syndromes^c^				
Childhood absence epilepsy	Paroxysms of 3 Hz (range = 2.5–4 Hz) generalized spike‐wave at the start of the absence^b^	Consistently unilateral epileptiform discharges Lack of HV‐activated 2.5–4 Hz generalized spike wave in untreated patient who performs HV well for 3 min or longer Recording a typical staring spell without EEG correlate in a child with a history of 2.5–4 Hz generalized spike wave Persistent slowing of the EEG background in the absence of sedating medication	Diffuse background slowing	4–10 years (range 2–13 years)
Juvenile absence epilepsy	Paroxysms of 3–5.5 Hz generalized spike‐wave^b^	Lack of HV‐activated 3–5.5 Hz generalized spike‐wave in an untreated patient who performs HV well for 3 min or longer Persistent EEG background slowing in the absence of a sedating medication	Consistently unilateral focal epileptiform discharges Diffuse background slowing Recorded typical staring spell without EEG correlate	9–13 years (range 8–20 years)
Juvenile myoclonic epilepsy	3–5.5 Hz generalized spike‐wave or generalized polyspike‐wave on EEG^b^	–	Habitual myoclonic event captured on EEG in the absence of polyspike– and spike–wave discharge Focal slowing Consistently unilateral focal epileptiform abnormalities Generalized slow spike wave at frequency <2.5 Hz (unless it is at the end of a higher frequency burst) Diffuse background slowing that is not limited to the postictal period	10–24 years (range: 8–40 years)
Epilepsy with generalized tonic–clonic seizures alone	3–5.5 Hz generalized spike‐wave or polyspike‐wave on EEG (may be obtained historically)	–	Focal slowing Consistently unilateral focal epileptiform discharges Generalized slow spike –wave at frequency <2.5 Hz (unless it is at the end of a higher frequency burst) Diffuse background slowing that is not limited to the postictal period	10–25 years (range: 5–40 years)
Epilepsy syndromes with onset at a variable age^d^				
Rasmussen syndrome	Hemispheric slowing Epileptiform abnormalities maximally over the affected hemisphere	Generalized spike‐and‐wave	–	1–10 years, it can rarely start in adolescent and adult years (≤10%)
Progressive myoclonus epilepsies	Generalized spike/polyspike‐wave	–	Persistent focal epileptiform abnormality, other than occipital	2–50 years; etiology dependent Onset at >20 years is an alert criterion
Epilepsy syndromes with onset in childhood^e^				
Self‐limited epilepsy with centrotemporal spikes	High amplitude, centrotemporal biphasic epileptiform abnormalities	Sustained focal slowing not limited to the postictal phase Persistently unilateral centrotemporal abnormalities on serial EEGs Lack of sleep activation of centrotemporal abnormalities	–	4–10 years (range: 3–14 years)
Self‐limited epilepsy with autonomic features	High‐amplitude, focal or multifocal epileptiform abnormalities that increase in drowsiness and sleep	Sustained focal slowing not limited to the postictal phase Unilateral focal abnormalities in a consistent focal area across serial EEGs	–	3–6 years (range: 1–14 years)
Childhood occipital visual epilepsy	Occipital spikes or spike‐wave abnormalities (awake or sleep)	Sustained focal slowing not limited to the postictal phase	–	8–9 years (range: 1–19 years)
Photosensitive occipital lobe epilepsy	Occipital epileptiform abnormalities facilitated by eye closure and IPS	Sustained focal slowing not limited to the postictal phase Photoparoxysmal response at slow photic frequency (1–2 Hz; suggest CLN2 disease)	–	4–17 years (range: 1–50 years)
Epilepsy with eyelid myoclonia	Eye closure and intermittent photic stimulation elicits fast (3–6 Hz) generalized polyspikes or polyspike‐wave complexes	–	Focal slowing Consistently unilateral focal spikes Generalized slow spike‐wave pattern at frequency <2.5 Hz (unless it is at the end of a higher frequency burst) Diffuse background slowing that is not limited to the postictal period Lack of EEG correlate with typical clinical event	6–8 years (range: 2–14 years)
Epilepsy with myoclonic absences	Regular 3 Hz generalized spike‐wave pattern time‐locked with myoclonic jerks	–	Focal slowing Consistently unilateral focal spikes Generalized slow spike‐wave pattern at frequency <2 Hz (unless it is at the end of a higher frequency burst) Diffuse background slowing that is not limited to the postictal period	Peak at 7 years (range: 1–12 years)
Epilepsy with myoclonic‐atonic seizures	Generalized 2–6 Hz spike wave or polyspike‐wave abnormalities	Generalized paroxysmal fast activity in sleep Generalized slow spike‐wave complexes of <2 Hz Photoparoxysmal response at low frequencies (suggests *CLN2* disease)	Persistent focal abnormalities Hypsarrhythmia	2–6 years (range: 6 months to 8 years)
Lennox–Gastaut syndrome	Generalized slow spike‐wave complexes of <2.5 Hz (or history of this finding on prior EEG) Generalized paroxysmal fast activity in sleep (or history of this finding on prior EEG)	Photoparoxysmal response at low frequencies (consider *CLN2* disease)	Persistent focal abnormalities without generalized spike‐wave pattern	Peak at 3–5 years; usually between 18 months and 8 years Onset in the 2nd decade is rare Onset must be at <18 years
Developmental and epileptic encephalopathy with spike‐and‐wave activation in sleep (SWAS) and epileptic encephalopathy with SWAS	Slow (1.5–2 Hz) spike‐wave abnormalities in non‐REM sleep Abnormalities are markedly activated in sleep	Generalized paroxysmal fast activity in sleep (consider Lennox–Gastaut syndrome) Generalized slow spike‐wave complexes of <2.5 Hz in both awake and asleep states (consider Lennox–Gastaut syndrome)	–	2–12 years (peak 4–5 years); it can rarely develop between 1 and 2 years
Febrile infection‐related epilepsy syndrome	Slowing of the background activity Multifocal epileptiform abnormalities Frequent, focal electrographic and electroclinical seizures	Unifocal seizures	–	2–17 years, exceedingly rare before 2 years, rare between 17 and 30 years
Hemiconvulsion‐hemiplegia–epilepsy syndrome	Slowing of background activity over the affected hemisphere Focal or multifocal epileptiform abnormalities over the affected hemisphere in the chronic phase	–	–	<4 years; it can rarely start between 4 and 6 years
Epilepsy syndromes with onset in infancy^f^				
Myoclonic epilepsy in infancy	Normal background *Generalized discharges in the form of spike‐wave or, less frequently, polyspike‐wave may be seen* (*and are more common in the early stages of sleep*)	Lack of generalized spike–wave discharges on sleep recording Photoparoxysmal response at low‐frequency photic stimulation (suggest *CLN2* disease)	Recorded myoclonic event without EEG correlate Hypsarrhythmia Generalized slow spike‐wave (<2.5 Hz)	4 months–3 years
Early infantile developmental and epileptic encephalopathy	Burst‐suppression or multifocal discharges or Diffuse background slowing	–	–	0–3 months (adjusted for prematurity)
Epilepsy of infancy with migrating focal seizures	Ictal recording shows a migrating seizure pattern (this might be missed if a prolonged video EEG is not performed) Interictal multifocal discharges	Suppression burst pattern prior to medication Single persistent epileptic focus on EEG Hypsarrhythmia	–	<6 months (mean 3 months); it can rarely start between 6 and 12 months
Infantile epileptic spasms syndrome	Hypsarrhythmia Multifocal or focal epileptiform discharges (that might be seen quickly after the spasms onset)	Normal EEG or Burst‐suppression pattern	Normal EEG during recorded clinical events of suspected spasms	3–12 months (range: 1–24 months)

*Note*: Please note that there are additional syndromes not included in this table due to the lack of mandatory diagnostic EEG criteria. Please note that epilepsy syndromes with exclusive onset in the neonatal period were similarly excluded.

^a^ Alert criteria are absent in most cases but rarely can be seen. Their presence should result in caution in diagnosing the syndrome and consideration of other conditions.

^b^ Required only at the beginning of the burst, the discharge can slow down as it disappears.

^c^ Modified from: Hirsch et al.^50^

^d^ Modified from: Riney et al.^51^

^e^ Modified from: Specchio et al.^52^

^f^ Modified from: Zuberi et al.^54^

The point prevalence of IEDs in individuals without seizures is 1.74%, with higher prevalence observed in children (2.45%) as opposed to adults (0.93%), with a subsequent increase in the elderly (5.96%).^60^ However, these estimates may be affected by potential misinterpretation of normal EEG variants as epileptiform patterns, such as rhythmic midtemporal theta of drowsiness (RMTD) and wicket waves.

Importantly, IEDs may be present in patients without a history of seizures. Studies have identified epileptiform abnormalities in approximately 30% of children with ADHD who have never experienced seizures.^61^ Patients with autism spectrum disorder without epilepsy have a higher prevalence of IEDs (~15%)^62^. Due to their behavioral issues that include non‐epileptic staring spells and behavioral dysregulation, using scalp EEG may prove challenging to study this patient population.^63^ Patients with ADHD have a higher prevalence of focal epileptiform discharges compared with generalized IEDs.^61^ The prognostic value of detecting IEDs in individuals without a seizure history remains unclear.^60^

#### 3.1.2 Pathophysiology

**FIGURE 11:**
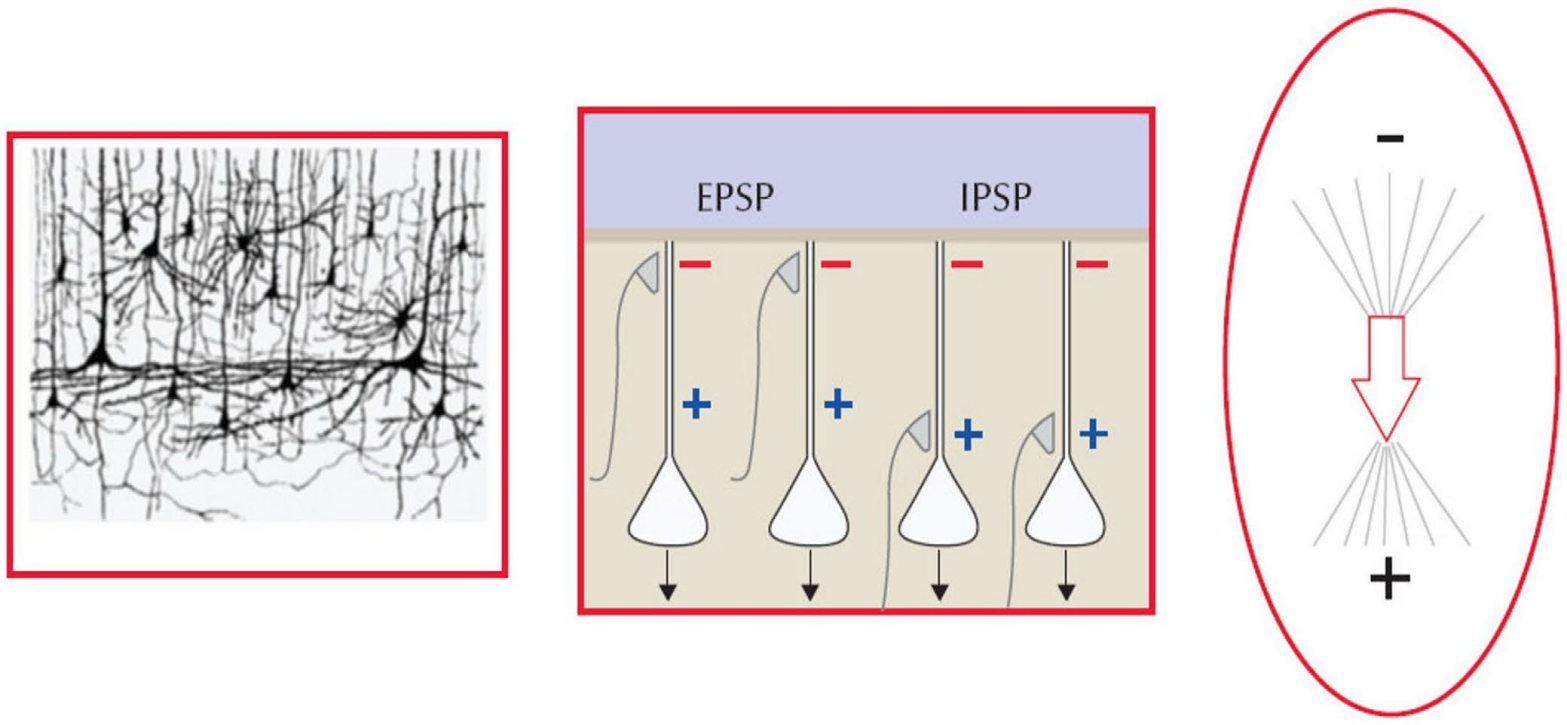
Schematic of cortical cytoarchitecture (left panel). Generation of excitatory postsynaptic potentials (EPSP) and inhibitory postsynaptic potentials (IPSP) (center panel)—in the case of ESPS, when there is excitation at dendrite level, the positive intracellular current flows into the nerve cell leaving a negative potential outside the cell near the cortical surface. In the case of IPSP, the negative potential seen in the cortical surface occurs due to a return current entering the apical dendrites thus creating extracellular negativity closer to the surface. Both superficial EPSP and deep IPSP functionally create the same surface negative dipole that characterizes IEDs (right panel). With permission, from Foged et al.^199^

Recognizing the distinct characteristics of IEDs is important for distinguishing them from normal variants and non‐epileptiform sharp transients (NESTs). IEDs are electrophysiological potentials seen on EEG that are produced by the summation of voltage field potentials generated by pyramidal cells in the cortex through a process known as paroxysmal depolarization shift (PDS). This process is characterized by synchronized changes in neuronal membrane potential, driven by the influx of calcium and sodium leading to action potential bursts and large excitatory postsynaptic potentials (EPSP).^56^ For IEDs to be detectable on scalp EEG, they require a cortical area of at least 10–20 cm^2^.^64^ Nonetheless, small generators, as small as 2–4 cm^2^, can produce scalp potentials comparable to larger ones, with factors such as generator location, skull thickness, cortical curvature, and background EEG activity influencing their detectability.^65^ The return currents generating IEDs have a dipole configuration, with negative polarity in the direction of the surface of the cortical generator, and positive polarity in the opposite direction (toward the white matter under the cortical generator), arising from the hypersynchronous depolarization of vertically oriented neurons (Figure 11).^66^ Radially oriented sources, with the generator located in the cortical convexity, typically have a negative polarity of larger amplitude, and these are easilyidentified. In contrast, IEDs with positive polarity are more often observed in intracranial EEG recordings,^67^ in neonates with intraventricular hemorrhage, or in patients with traumatic brain injury or skull defects.^66^, ^67^ In tangentially oriented dipoles, where the direction of the current flow is parallel with the surface, the maximum surface negativity shifts away from the electrode recording above the region due to the orientation of the cortical generator. Hence, tangential dipoles are better localized with inspection of the voltage maps or source localization strategies.^66^ Most IEDs are not specific to the underlying etiology of epilepsy. However, a notable exception is the association between nearly continuous repetitive sharp waves or spikes and underlying focal cortical dysplasia (FCD).^68^

Several factors influence the detection of IEDs on routine EEG, such as state of consciousness, baseline frequency of seizures, patient age, time since the last seizure, and duration of EEG recording.^29^, ^69^ IED detection has been shown to increase during periods of NREM sleep compared with wakefulness. Sleep deprivation can significantly enhance the detection of epileptiform abnormalities by evoking sleep and enabling recording during the wake–sleep transition. Certain procedures, like hyperventilation and photic stimulation, can trigger epileptiform activity primarily in genetic generalized epilepsies (GGEs), especially in the subgroup of idiopathic generalized epilepsies (IGEs), such as childhood absence epilepsy (CAE) and juvenile myoclonic epilepsy (JME).^50^, ^70^ Although such activation is uncommon in focal epilepsies, an increased rate of spike detection may be seen in patients with occipital onset epilepsy.^71^ Despite these techniques, scalp EEGs have limitations in sensitivity for IED detection. Often, spikes detected via subdural or stereo‐electroencephalography electrodes are undetected on scalp EEG, especially when less than 10 cm^2^ of cortical area is involved in their generation.^64^ Factors such as the area and depth of the cortical source and the orientation of the epileptiform activity source can influence IED detection on scalp EEG.^72^ Furthermore, the first routine EEG recording identifies IEDs in 29%–55% of cases, with repeat routine EEGs increasing the yield to approximately 90%.^73^ Additionally, the correlation between IED location and epilepsy risk varies, with central‐mid‐temporal (40%) and occipital (50%) spikes having the lowest association, while anterior temporal, midtemporal, and multifocal spikes being are strongly linked to epilepsy (90%–95%).^74^

#### 3.1.3 International Federation of Clinical Neurophysiology criteria for IEDs and their types

**FIGURE 12:**
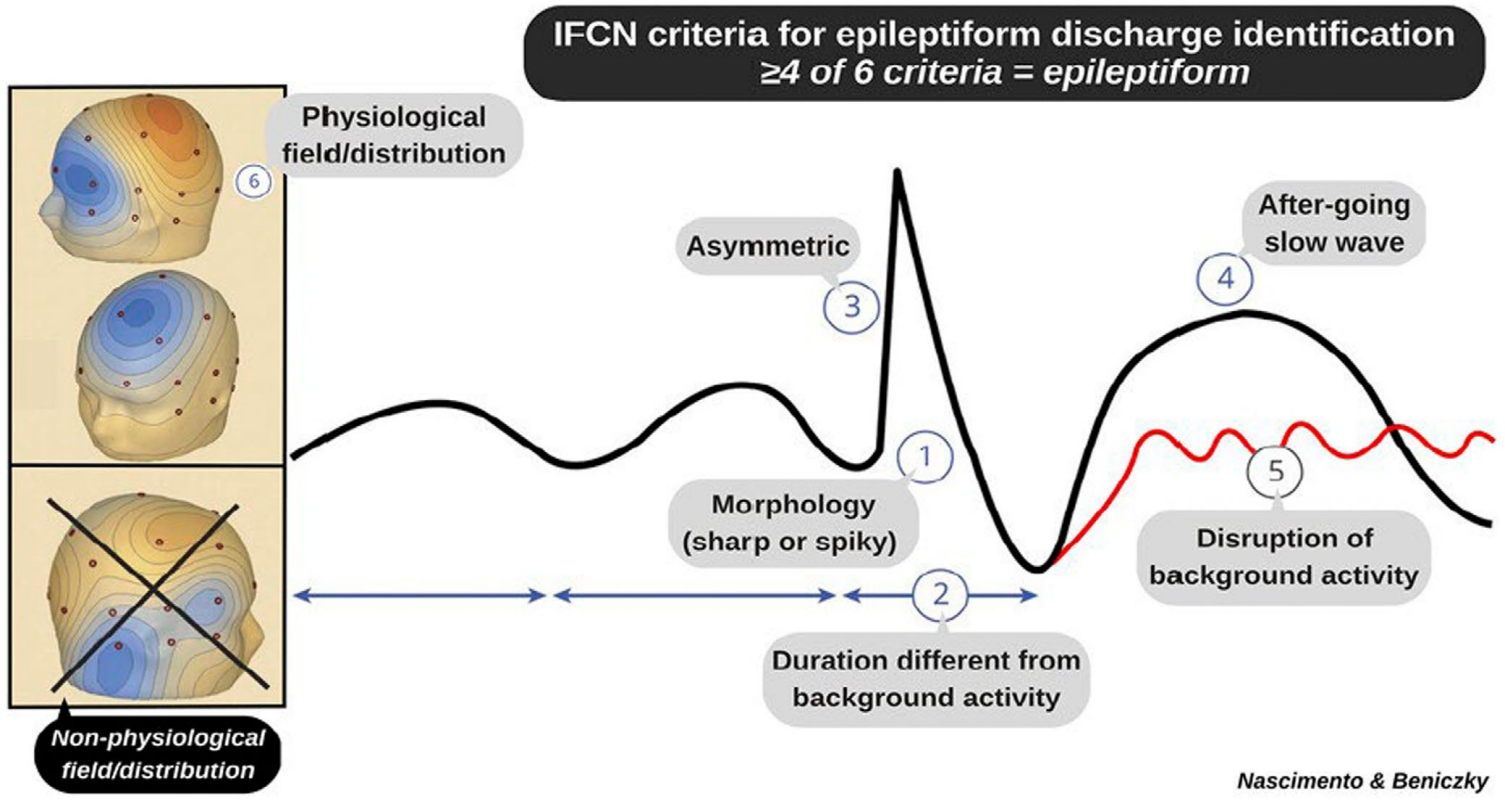
International Federation of Clinical Neurophysiology criteria for epileptiform discharge identification: (1) sharp or spiky morphology (20–200 ms); (2) different wave duration than background activity; (3) waveform asymmetry; (4) after‐going slow wave; (5) disruption of background activity: Flattening or low‐voltage alpha or beta frequency activity after (most frequently) or before sharp transient; (6) distribution suggestive of cerebral source/physiologic field. Criteria 4 and 5 are independent. IFCN, International Federation of Clinical Neurophysiology. With permission from: Nascimento F, Beniczky S. 2023, Neurology.

Misinterpretation of EEG often leads to the misdiagnosis of epilepsy.^4^ Studies show that there is inconsistency among experts in identifying IEDs on EEG.^75^, ^76^, ^77^, ^78^ To address this variability, the International Federation of Clinical Neurophysiology (IFCN) introduced six criteria for accurately recognizing IEDs on EEG. It is recommended that at least four of these six criteria be met to classify a sharp transient as an IED (Figure 12). Notably, these criteria apply to a single occurrence of the transient, without considering the significance of the quantity or frequency of the discharge within the recording.^79^ It has been shown that when only four criteria are identified, a higher rate of occurrence is needed (at least four times in a routine EEG recording) to avoid over‐reading.^80^

#### 3.1.4 IFCN criteria

1. Di‐ or triphasic waves with sharp or spiky morphology.—REMEMBER: *Pointy*
2. Different waveform duration compared with the ongoing background activity, either shorter or longer.—REMEMBER: *Different duration*
3. Asymmetry of the waveform: a sharply rising ascending phase and a more slowly decaying descending phase, or vice versa.—REMEMBER: *Asymmetric*
4. The transient is followed by an associated slow after‐wave.—REMEMBER: After‐going *slow wave*
5. The background activity surrounding the sharp transient is disrupted by the presence of the transient.—REMEMBER: *Disrupts the background*
6. Distribution of the negative and positive potentials on the scalp suggests a source of the signal in the brain corresponding to a radial, oblique, or tangential orientation of the source. This can be assessed by inspecting voltage maps constructed using common average reference.—REMEMBER: *Field makes sense*

#### 3.1.5 IEDs are classified by their morphology and duration

##### Spike

**FIGURE 13:**
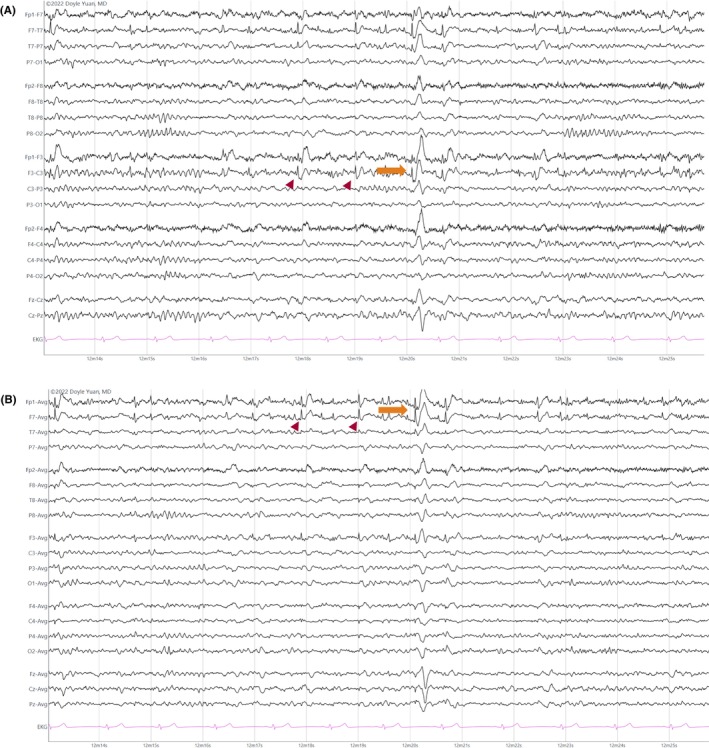
Focal spikes (arrowheads) and spike–wave (orange arrow) discharges in the left frontal region in a 15‐year‐old female. Panel (A): bipolar longitudinal montage; panel (B): common average referential montage. Low‐frequency filter 1 Hz, high‐frequency filter 70 Hz, sensitivity 7 μV/mm for panel A and 10 μV/mm for panel B.

The pointiest epileptiform discharge, with a very steep upslope and downslope lasting 20–70 msec (Figure 13).^56^

##### Polyspikes

**FIGURE 14:**
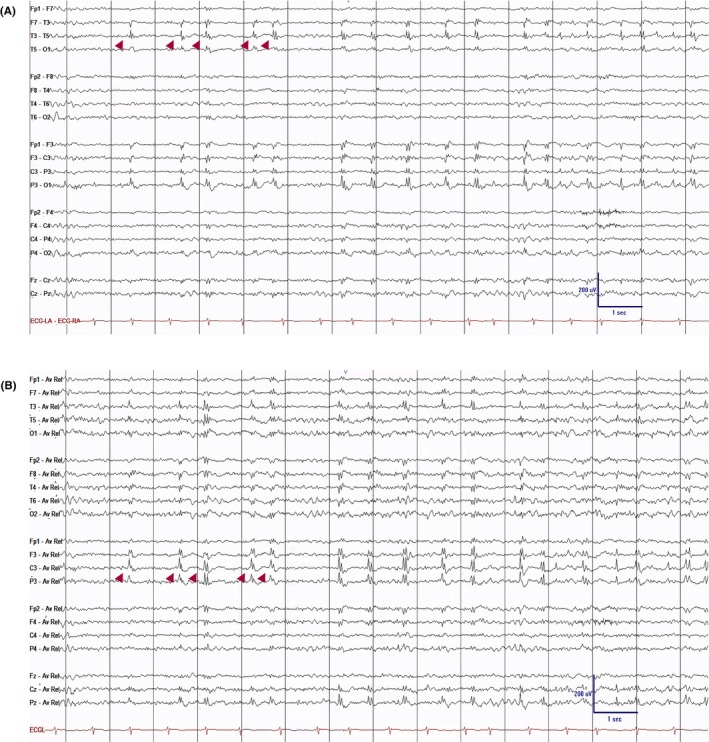
Focal polyspikes in the left centroparietal region (arrowheads) at times occurring periodically at a frequency of 1–2 Hz in a 61‐year‐old male with left precentral gyrus focal cortical dysplasia. Panel (A): bipolar longitudinal montage; panel (B): common average referential montage. Low‐frequency filter 1 Hz, high‐frequency filter 70 Hz, sensitivity 10 μV/mm.

A sequence of two or more consecutive spikes lasting <0.5 s is termed polyspikes.^10^, ^81^ (Figure 14).^68^, ^82^

##### Sharp wave

**FIGURE 15:**
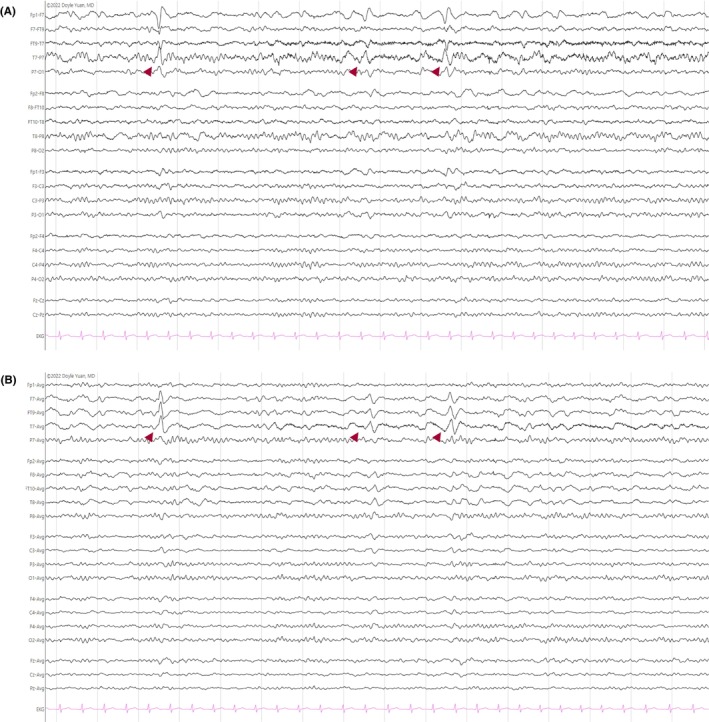
Left temporal sharp waves (arrowheads) in a 17‐year‐old patient with new‐onset seizures. Panel (A): bipolar longitudinal montage; panel (B): common average referential montage. Low‐frequency filter 1 Hz, High‐frequency filter 70 Hz, sensitivity 10 μV/mm.

Pointed peak with less steep upslope and downslope lasting 70–200 msec (Figure 15).^56^

##### Spike‐wave complex or polyspike‐wave complex or sharp‐and‐wave complex

**FIGURE 16:**
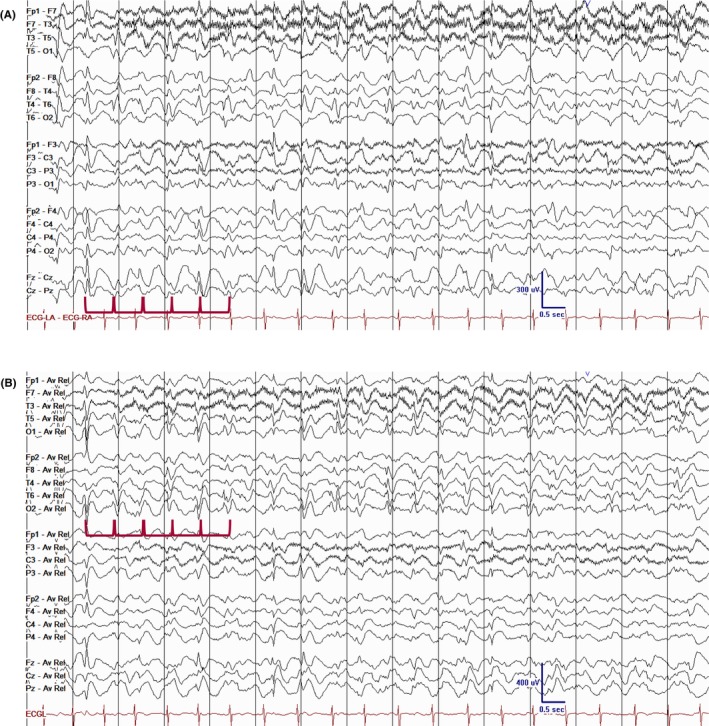
Generalized slow (<2.5 Hz) spike–wave discharges (brackets) in a 22‐year‐old patient with Lennox–Gastaut syndrome. Panel (A): bipolar longitudinal montage; panel (B): common average referential montage. Low‐frequency filter 1 Hz, high‐frequency filter 70 Hz, sensitivity 15 μV/mm for panel A and 20 μV/mm for panel B.

**FIGURE 17:**
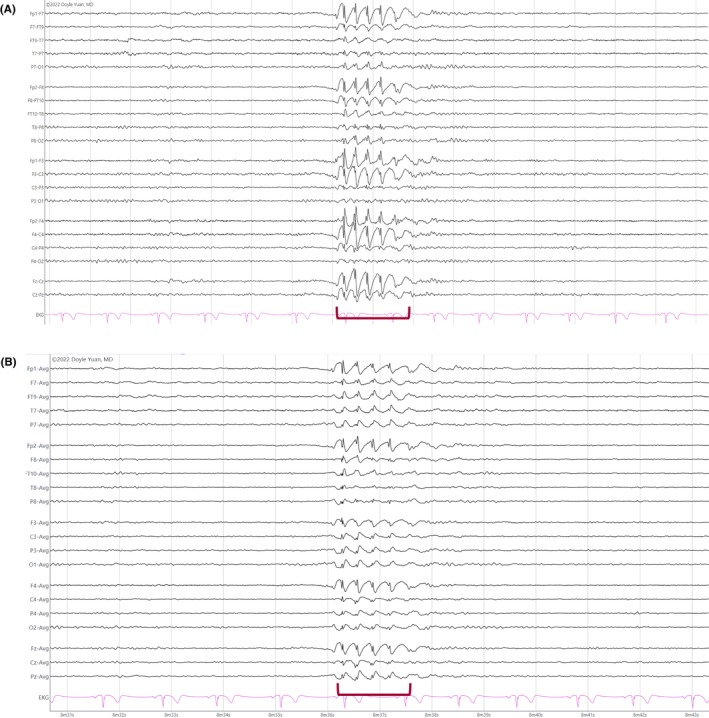
Generalized 3 Hz spike–wave discharge burst lasting 2 seconds with bilateral frontocentral predominance (bracket) in an 18‐year‐old patient. Panel (A): bipolar longitudinal montage; panel (B): common average referential montage. Low‐frequency filter 1 Hz, high‐frequency filter 70 Hz, sensitivity 25 μV/mm for panel A and 50 μV/mm for panel B.

**FIGURE 18:**
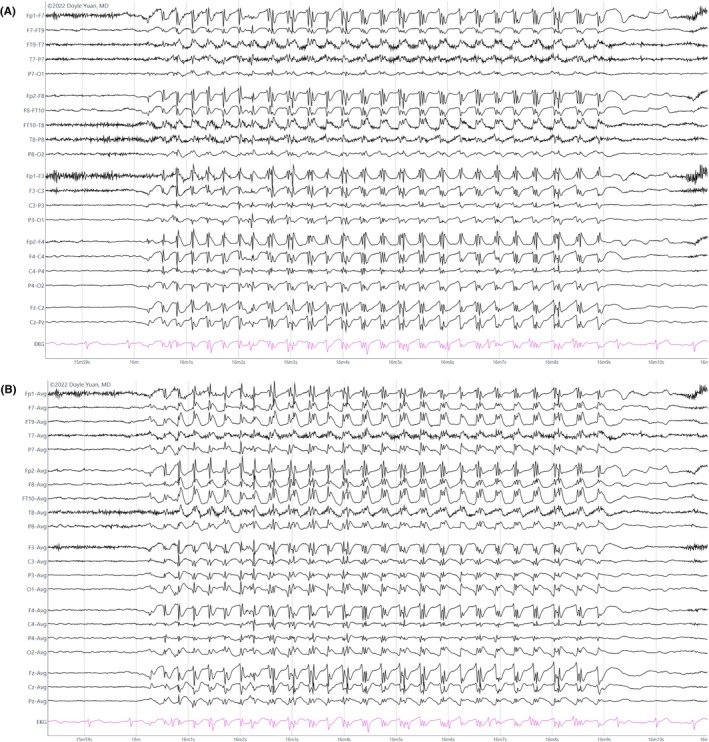
Generalized 3.5–4 Hz polyspike–wave discharges in a 14‐year‐old patient with juvenile myoclonic epilepsy (JME). Panel (A): bipolar longitudinal montage; panel (B): common average referential montage. Low‐frequency filter 1 Hz, high‐frequency filter 70 Hz, sensitivity 50 μV/mm.

These are epileptiform discharges characterized by either a spike, polyspike, or a sharp wave immediately followed by a slow wave that clearly stands out from the background activity. These may occur in isolation or in trains of multiple complexes, and may be focal or generalized. When generalized, the frequency observed after the first second of the burst—once the discharges are “established”—may point to specific etiologies/epilepsy syndromes. Some of the commonly encountered epilepsy syndromes are listed below (also refer to Table 4)^49^, ^83^:

- ≤2.5 Hz generalized spike–wave discharges—developmental and/or epileptic encephalopathies (DEEs), such as Lennox–Gastaut syndrome (LGS), (Figure 16).
- 3–4 Hz generalized spike–wave discharges—CAE, juvenile absence epilepsy (JAE) (Figure 17).
- 4–5 Hz generalized spike/polyspike–wave discharges—JME (Figure 18)

##### Paroxysmal fast activity (PFA)

**FIGURE 19:**
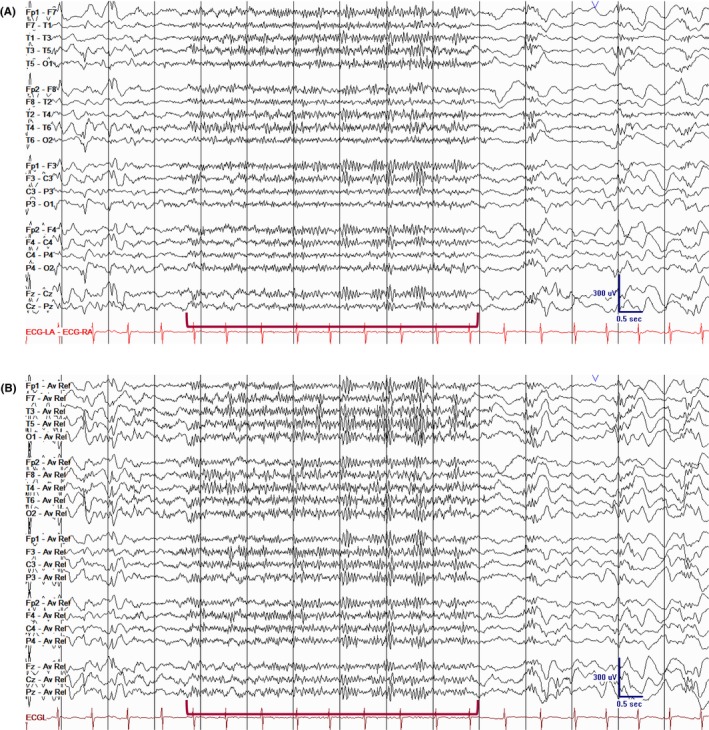
Generalized paroxysmal fast activity (GPFA) (bracket) in a 22‐year‐old patient with Lennox–Gastaut syndrome. Panel (A): bipolar longitudinal montage; panel (B): common average referential montage. Low‐frequency filter 1 Hz, high‐frequency filter 70 Hz, sensitivity 15 μV/mm.

PFA is characterized by a burst of low‐amplitude spikes (run of rapid spikes) usually lasting ≥300 msec that can occur focally (focal PFA) or more broadly (generalized PFA), at frequencies of 10–25 Hz (typically around 10 Hz) (Figure 19).^84^, ^85^ Focal PFA has been reported in patients with FCD and gliosis and may be seen in up to 25% of patients with pharmacoresistant epilepsy.^84^ Generalized paroxysmal fast activity (GPFA) has a duration of 2–10 s as per the IFCN,^43^ and is commonly observed during sleep in both pharmacoresponsive and pharmacoresistant epilepsies. It has been reported most often in patients with LGS, but also may be seen, though infrequently, in typical absence seizures and epilepsy with eyelid myoclonia (EEM; formerly known as Jeavons syndrome).^85^ These usually have a high amplitude exceeding 100 μV and have been found to be more common in females.^85^, ^86^

#### 3.1.6 Other epileptiform and potentially epileptiform abnormalities

##### Hypsarrhythmia

**FIGURE 20:**
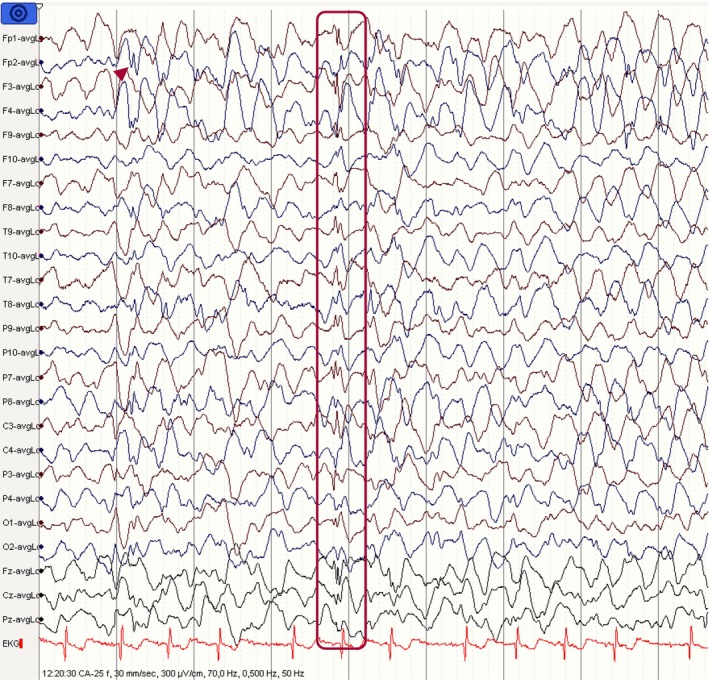
Hypsarrhythmia in an infant with epileptic spasms. Note the exceedingly high amplitude of the waveforms as well as focal (arrowhead) and generalized (rectangle) epileptiform discharges. Common average referential montage. Low‐frequency filter 0.5 Hz, high‐frequency filter 70 Hz, sensitivity 300 μV/mm.

Hypsarrhythmia, first described by Gibbs and Gibbs in 1952, is a hallmark interictal EEG pattern frequently associated with infantile epileptic spasms^54^, ^87^, ^88^, although lack of hypsarrhythmia does not exclude the diagnosis of infantile epileptic spasms syndrome.^89^ This EEG signature is characterized by diffuse, high‐amplitude (>200 μV), disorganized slow waves with multifocal epileptiform discharges, typically affecting both cerebral hemispheres and most often seen in sleep (Figure 20)^90^. Due to poor inter‐rater reliability (IRR) in determining hypsarrhythmia, an interictal EEG grading scale designed to improve diagnostic consistency named the *Burden of Amplitude and Epileptiform Discharges* (BASED) score was developed. This score assesses the severity of interictal EEG abnormalities by grading the presence and extent of epileptiform discharges, assessing spike foci, and amplitudes^91^, ^92^ Although less common, this pattern may occur asymmetrically or unilaterally. Distinct variants of hypsarrhythmia can be seen: hypsarrhythmia with increased interhemispheric synchronization characterized by more coordinated activity across hemispheres, and hypsarrhythmia patterns with a consistent focus of epileptiform activity associated with localized spike‐ or sharp‐wave activity. Additionally, some variants are associated with periods of voltage attenuation.^93^

### 3.2 Focal abnormalities, other than IEDs

#### 3.2.1 Focal slowing

**FIGURE 21:**
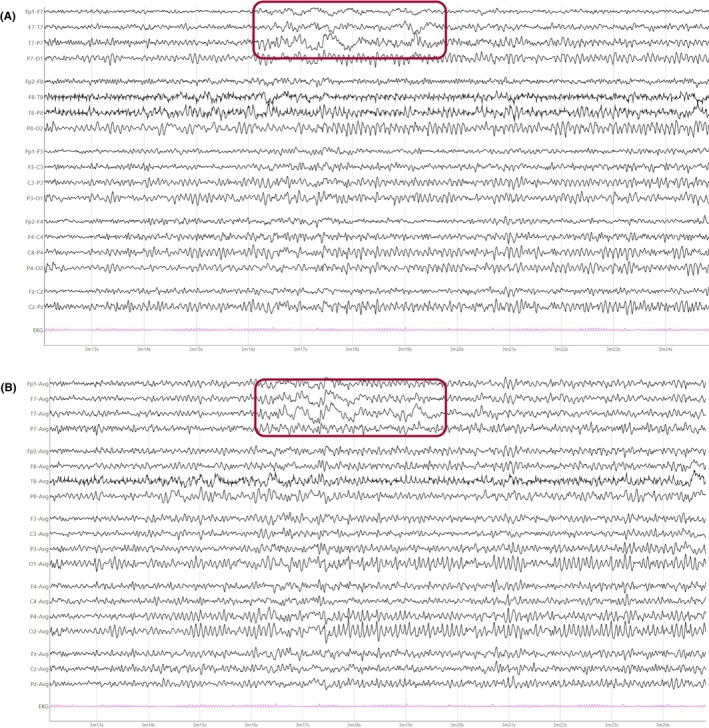
Focal polymorphic delta slowing in the left temporal region (rectangle) in a 52‐year‐old male with events of dysarthria and transient right facial weakness. Panel (A): bipolar longitudinal montage; panel (B): common average referential montage. Low‐frequency filter 1 Hz, high‐frequency filter 70 Hz, sensitivity 7 μV/m.

**FIGURE 22:**
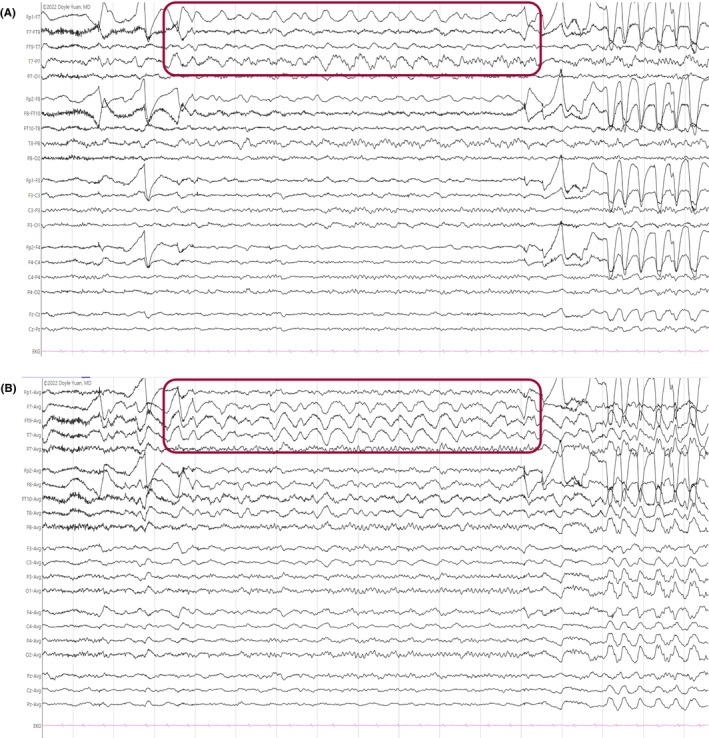
Left temporal intermittent rhythmic delta activity (TIRDA) (rectangle) as seen in a 17‐year‐old patient with new‐onset seizures. Panel (A): bipolar longitudinal montage; panel (B): common average referential montage. Low‐frequency filter 1 Hz, high‐frequency filter 70 Hz, sensitivity 10 μV/mm.

Focal slowing is characterized by the presence of waveforms in the delta (.5–4 Hz) or theta (4–8 Hz) frequency range usually restricted to a single region of the brain (Figure 21). Focal slowing with an irregular shape (polymorphic) is a common abnormal EEG finding. When present continuously, focal slowing has a high likelihood of being associated with a structural lesion^94^; however, it does not provide precise information about the type of lesion.^95^ A variety of structural abnormalities, including but not limited to tumors,^96^ vascular lesions,^97^, ^98^ traumatic brain injuries,^99^ infections,^100^ FCDs,^101^ and mesial temporal sclerosis^95^ can exhibit this pattern. Focal slowing may also occur in the absence of a discernible structural lesion in the brain. For example, it can be observed in non‐lesional focal epilepsies,^102^ postictal state,^103^ and cerebrovascular diseases that alter blood flow to the brain, such as vasospasm.^104^ Moreover, focal slowing without an underlying structural lesion has been documented in individuals with a history of migraines.^105^ The pathophysiologyic behind polymorphic delta slowing remains poorly understood. These abnormalities likely originate from deafferented cortical areas, due to white matter abnormalities affecting the cholinergic input to the cortex they target.^106^, ^107^ It has been shown in animal studies that lesions limited to the cortex cause attenuation of faster frequencies on the background without slow‐wave intrusion, while subcortical white matter lesions deafferented from the cortex produce polymorphic delta activity, and combined cortical–subcortical lesions suppress faster rhythms with delta activity.^108^ In patients with traumatic brain injuries, disruption of the blood–brain barrier has been proposed as the likely mechanism.^99^ When temporal focal delta slowing occurs rhythmically, it is called temporal intermittent rhythmic delta activity (TIRDA). Over 40% of patients with TIRDA on EEG have temporal lobe epilepsy (Figure 22).^109^ Therefore, TIRDA is considered an epileptiform equivalent.^110^ A significant proportion of patients with this EEG pattern also show signal abnormalities in mesial temporal structures on neuroimaging, with confirmed pathological findings of mesial temporal sclerosis in several cases.^109^

#### 3.2.2 Asymmetry

**FIGURE 23:**
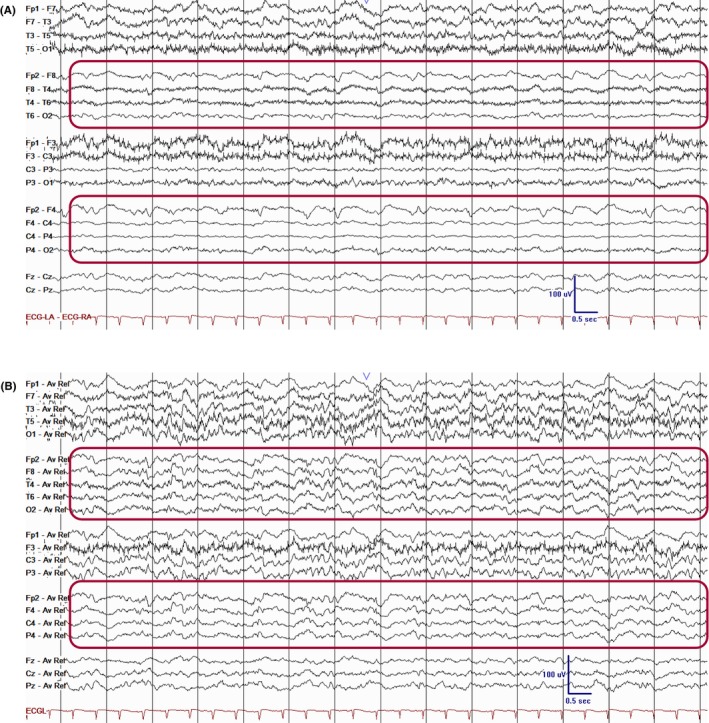
Asymmetry with voltage attenuation and relative loss of faster frequencies on the right hemisphere (rectangles) in a 55‐year‐old male with right middle cranial fossa/skull base tumor. Panel (A): bipolar longitudinal montage; panel (B): common average referential montage. Low‐frequency filter 1 Hz, high‐frequency filter 70 Hz, sensitivity 7 μV/mm.

The term “asymmetry” refers to a difference in amplitude relative to the baseline background rhythm or a difference in frequency between the two hemispheres.^10^ This is best assessed using referential montages, as bipolar montages may be affected by in‐phase cancellation between adjacent electrodes. EEG background can be described as symmetric, mildly asymmetric, or markedly asymmetric. Mild asymmetry is described as a difference in amplitude of <50% when measured in a referential montage or a consistent difference in frequency of 0.5 to 1 Hz between the two hemispheres. A difference in amplitude of ≥50% or >1 Hz difference in frequency is considered as marked asymmetry.^10^ Notably, a mild degree of asymmetry in amplitude is often present in normal EEG recordings. For instance, the PDR in normal EEG usually displays a lower amplitude on the left side compared with the right, as discussed above.^111^ A reduction in amplitude of approximately ≥50% difference in frequency is deemed substantial enough to be considered abnormal (Figure 23). Asymmetry is often a sign of focal dysfunction on the side where the amplitude or frequency is reduced.^112^ An exception to this rule is what is known as breach rhythm. This phenomenon occurs due to a defect in the skull, which acts as the strongest resistance element between the brain and the recording electrode, and, therefore, its absence increases the amplitude of the activity recorded at or near the defect.^113^ When skull breach is present, the faster frequencies have higher amplitude on the side of the defect and, as a result, often appear sharply contoured.^10^ Similar to focal slowing, asymmetry is not specific to any etiology but is usually indicative of an underlying structural brain lesion, suggesting focal dysfunction.

### 3.3 Generalized non‐epileptiform abnormalities

#### 3.3.1 Generalized slowing

**FIGURE 24:**
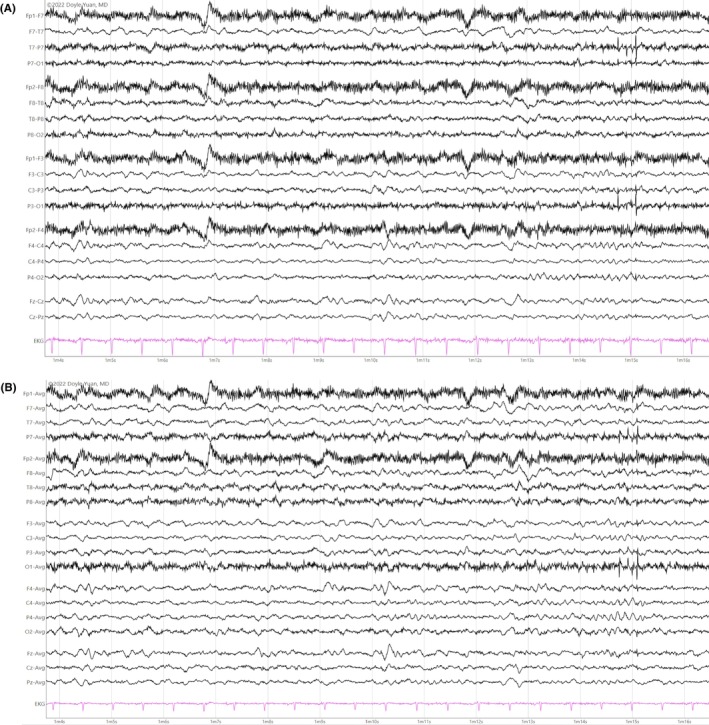
Generalized polymorphic (irregular, arrhythmic) delta slowing in a 67‐year‐old male admitted for delirium and agitation. Note the diffuse delta and theta waveforms and lack of clear anterior to posterior gradient and absence of a posterior dominant rhythm. Panel (A): bipolar longitudinal montage; panel (B): common average referential montage. Low‐frequency filter 1 Hz, high‐frequency filter 70 Hz, sensitivity 7 μV/mm.

The presence of generalized delta activity (0.5–4 Hz) outside of slow‐wave sleep (N3) and theta activity (4–8 Hz) outside of drowsiness and light sleep (N1 and N2) is considered abnormal in an adult EEG. These findings are most commonly seen in patients with encephalopathy (Figure 24). Generalized slowing can be characterized by its morphology, which may be either polymorphic (irregular or arrhythmic) or monomorphic (regular or rhythmic). Generalized slowing has been observed in several processes, such as traumatic brain injury, neurodegenerative conditions, neurodevelopmental delay, toxic or metabolic encephalopathies, meningoencephalitis, and the effect of sedative medications.^114^

Generalized irregular theta slowing correlates with a milder degree of encephalopathy compared with generalized irregular delta slowing, especially in the setting of background variability and reactivity.^107^ Generalized irregular slowing can be seen intermittently or continuously, usually as a mixture of slower frequencies with a predominance of either theta or delta frequencies. It has been suggested that the dominance of a single frequency, with little to no variability, may indicate a more severe degree of encephalopathy.^107^

#### 3.3.2 Generalized slowing with posterior predominance

**FIGURE 25:**
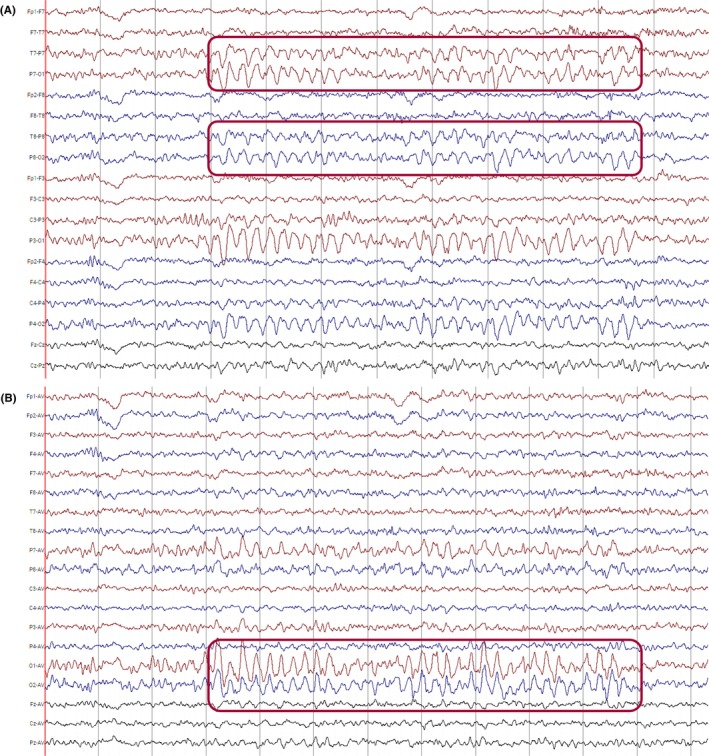
Occipital rhythmic delta activity (OIRDA) (red rectangles). Panel (A): bipolar longitudinal montage; panel (B): common average referential montage. Low‐frequency filter 1 Hz, high‐frequency filter 70 Hz, sensitivity 10 μV/mm.

In children, adolescents, and young adults, polymorphic posterior delta waves intermixed with the PDR may appear. These are known as posterior slow waves of youth (PSWY) and are considered a normal EEG variant.^115^ When posterior delta slowing becomes monomorphic and disrupts the PDR, it is described as occipital intermittent rhythmic delta activity (OIRDA)^116^. OIRDA occurs in about 0.2% to 0.7% of EEGs in children under the age of 10 years, and is characterized by synchronized, sinusoidal bursts of high‐amplitude activity at approximately 3 Hz (Figure 25)^117^. When seen in patients with epilepsy, OIRDA is most characteristically seen in CAE.^116^ This association is illustrated by the term coined by Aird and Gastaut: *slow posterior rhythm associated with petit mal* ^115^. OIRDA may also be found in focal epilepsy, albeit to a lesser extent^118^, as well as in patients with encephalopathy^119^. The exact pathogenesis is not well understood. Current theories suggest hyperexcitable thalamocortical (occipital) circuitry may play a role in this condition.^120^

### 3.4 EEG patterns in critically ill patients—The American Clinical Neurophysiology Society critical care EEG standardized critical care EEG terminology

With the advent of continuous EEG monitoring in the neurological intensive care setting, EEG readers have encountered a multitude of patterns commonly seen in the critically ill. To facilitate a common language for their report and for research purposes, the American Clinical Neurophysiology Society (ACNS) developed a standardized nomenclature.^10^ While it may be tempting to use this nomenclature in the reports of routine, ambulatory, or EMU EEG recordings, its use should generally be reserved for critically ill patients as these are fundamentally different patient populations, and the goal of EEG monitoring is often not the same. In the following section, we discuss some of the most common patterns encountered in critical care EEG.

#### 3.4.1 Generalized periodic discharges with triphasic morphology

Per the 2021 ACNS terminology, generalized periodic discharges (GPDs) with triphasic morphology—formerly known as “triphasic waves”—are a specific subtype of GPDs. Therefore, not all GPDs have a triphasic morphology.^10^

**FIGURE 26:**
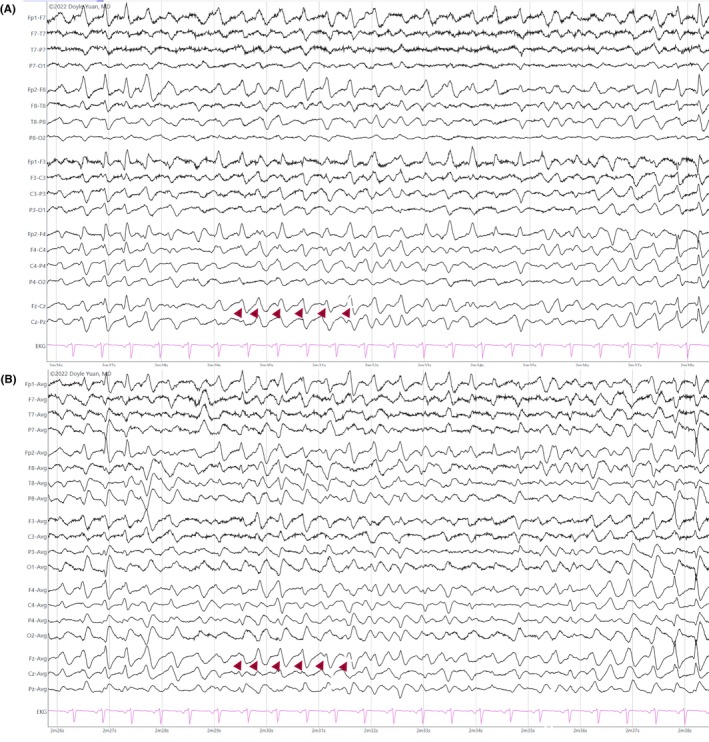
Generalized periodic discharges with triphasic morphology (arrowheads) occurring at a frequency of approximately 2.5 Hz in a 52‐year‐old male with liver cirrhosis and end‐stage renal disease. Panel (A): bipolar longitudinal montage; panel (B): common average referential montage. Low‐frequency filter 1 Hz, high‐frequency filter 70 Hz, sensitivity 7 μV/mm for panel A and 10 μV/mm for panel B.

GPDs with triphasic morphology are characterized by a negative deflection at onset, followed by a higher amplitude positive deflection at the peak (usually 150–250 μV), followed by a smaller negative deflection upon return to baseline (negative–positive–negative). The initial negative deflection is usually low voltage and may sometimes be obscured by ongoing background, giving a biphasic appearance (positive–negative) (Figure 26).^10^ This pattern is usually maximal anteriorly—though occasionally it is maximal posteriorly—in a bisynchronous periodic manner and is considered periodic only if it continues for at least six cycles.^10^, ^121^

GPDs with triphasic morphology were described by Foley et al.^122^ in 16 patients with hepatic encephalopathy. Bickford and Butt^123^ were the first to term this pattern as “triphasic waves.” In bipolar montages, GPDs with triphasic morphology often have a characteristic anterior to posterior (A‐P) lag, where the most positive point of the waveforms seems to move forward by a few milliseconds (25–140 msec) in each consecutive channel. Alternatively, the lag may be reversed (P‐A) or mixed; however, this characteristic lag usually disappears in referential montages (Figure 26).^121^ Typically, GPDs with triphasic morphology occur in bilaterally synchronous runs and are associated with mixed delta and theta background slowing.^124^

The pathophysiology behind the generation of GPDs with triphasic morphology remains largely unknown.^125^ Although these waves are commonly associated with hepatic and renal diseases, they can also manifest in other conditions, such as anoxia, sepsis, Hashimoto encephalopathy, and as a result of medication toxicity—baclofen and cefepime being the most remarkable examples.^126^, ^127^ Furthermore, GPDs with triphasic morphology have also been reported in patients with thalamic infarction(s), ifosphamide toxicity, intracranial hypertension, Angelman syndrome as well as with nonconvulsive seizures and nonconvulsive status epilepticus (NCSE).^128^, ^129^

#### 3.4.2 Generalized rhythmic delta activity

**FIGURE 27:**
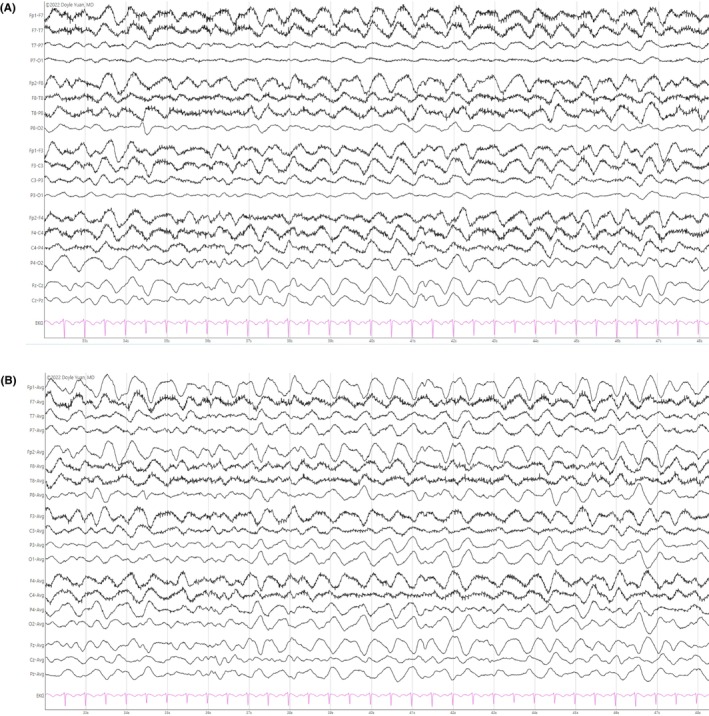
Generalized rhythmic delta activity (GRDA) with frontal predominance in a 34‐year‐old male. Panel (A): bipolar longitudinal montage; panel (B): common average referential montage. Low‐frequency filter 1 Hz, high‐frequency filter 70 Hz, sensitivity 7 μV/mm.

Generalized delta slowing can also be present in a regular, rhythmic pattern, referred to as generalized rhythmic delta activity (GRDA). This is characterized by generalized activity at less than 4 Hz that is monomorphic or regular, without intervals between consecutive waveforms, for at least six cycles. It may show a frontal, occipital, or midline predominance (Figure 27).^10^ Frontally predominant GRDA was previously known as frontal intermittent rhythmic delta activity (FIRDA). This term was initially given by van Der Drift and Magnus in 1961.^117^ Several studies initially linked the presence of GRDA to either increased intracranial pressure or the presence of a brain tumor.^130^, ^131^, ^132^ Later studies expanded the range of conditions associated with GRDA, including vascular lesions, infections, metabolic conditions, such as hepatic and renal insufficiency, and medication toxicity^117^ thus indicating that GRDA is a non‐specific EEG pattern. GRDA has also been shown to affect adults more than children and may appear in a wide range of medical conditions, highlighting its lack of specificity as a diagnostic tool.^133^ Importantly, GRDA is not considered a pattern that falls within the ictal–interictal continuum per the 2021 ACNS ICU EEG terminology.

#### 3.4.3 Lateralized periodic discharges

**FIGURE 28:**
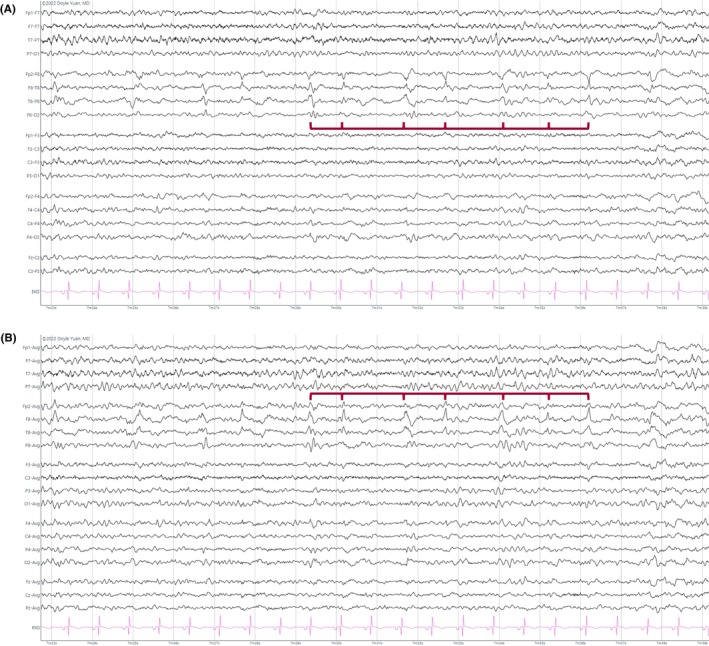
Lateralized periodic discharges (LPDs) in a 74‐year‐old female with an acute ischemic right middle cerebral artery stroke. See the periodic 1–1.5 Hz sharp waves occurring at F8 > T8 (brackets). Panel (A): bipolar longitudinal montage; panel (B): common average referential montage. Low‐frequency filter 1 Hz, high‐frequency filter 70 Hz, sensitivity 10 μV/mm.

The understanding of lateralized periodic discharges (LPDs) on EEG has evolved significantly since the initial report of a “periodic” EEG pattern in 1950.^134^ In 1964, the term “periodic lateralized epileptiform discharges” (PLEDs) was introduced to describe sharp waves that appeared at regular intervals in series of 33 patients.^135^ The ACNS later revised the terminology, replacing PLEDs with LPDs to reflect the uncertain relationship between these patterns and epileptic seizures by removing the term “epileptiform.” LPDs are characterized by “repetition of a (lateralized) waveform with relatively uniform morphology and duration, a clearly discernible inter‐discharge interval between consecutive waveforms, and the recurrence of the waveform at nearly regular intervals.” A pattern is defined as periodic if it continues for at least six cycles.^10^ LPDs are commonly seen in critically ill patients, where they are markers of a structural lesion and indicate a heightened predisposition to seizures, such as in brain tumors, subdural hematomas, subarachnoid hemorrhage, brain abscesses, cerebral infarctions, and infections like herpes simplex virus encephalitis. Notably, ischemic stroke is considered the most common cause of LPDs (Figure 28).^134^ The designation “LPDs plus” was introduced to describe LPDs with additional features, such as superimposed fast (+F) or rhythmic activity (+R).^10^ LPDs are considered to fall within ictal–interictal continuum (IIC), which means they are considered as potentially ictal if they meet the following criteria: (1) average frequency > 1.0 and ≤2.5 Hz over 10 s or (2) average frequency ≥ 0.5 Hz and ≤1.0 Hz over 10 s, including a plus modifier or fluctuation.^10^ While their pathophysiology remains unclear, LPDs reflect synchronous neuronal firing resulting from abnormal neuronal responses to acute brain injuries involving cortex, thalamocortical neurons, or both, emphasizing the complexity of their underlying mechanisms.^134^

#### 3.4.4 Lateralized rhythmic delta activity

Lateralized rhythmic delta activity (LRDA), as outlined by the ACNS terminology guidelines, represents a specific EEG pattern characterized by monomorphic (rhythmic) delta activity that continues for at least six cycles and may have different appearances, such as blunt sinusoidal or sharply contoured waves.^136^ The frequency of LRDA typically ranges from 1 to 2 Hz. It is important to differentiate LRDA from polymorphic delta slowing to ensure accurate interpretation and diagnosis in the clinical setting. Unlike focal polymorphic delta slowing, LRDA oftentimes suggests a greater likelihood of developing focal seizures. For example, 63% of critically ill patients who have LRDA during continuous EEG monitoring experienced seizures, suggesting a strong correlation between LRDA and a low seizure threshold.^137^ Most patients with LRDA have been noted to have abnormalities in the cerebral cortex, white matter, and/or deep gray nuclei.^117^

#### 3.4.5 Brief potentially ictal rhythmic discharges

Brief potentially ictal rhythmic discharges (BIRDs) are runs of focal or generalized rhythmic activity >4 Hz (at least six waves at a regular rate), lasting 0.5 to <10 seconds, that are not consistent with a known normal pattern or benign variant, and have no clinical correlation. If BIRDs evolve (see below section on ictal findings) or have a similar morphology and location to IEDs or seizures seen in the same patient, they are considered definite BIRDs. If they do not fulfill those criteria, but are sharply contoured in morphology, they are considered possible BIRDs.^10^ BIRDs can occur in critically ill and non‐critically ill patients and are associated with a significantly increased risk of seizures. Experts consider BIRDs to share the same pathophysiology as seizures, which have arbitrarily been defined as requiring a duration of at least 10 seconds by the ACNS for the purpose of standardizing the definition.

## 4 ICTAL FINDINGS

### 4.1 Introduction

According to the ILAE, a seizure is defined as the transient occurrence of signs and/or symptoms due to abnormal excessive or synchronous neuronal brain activity.^138^ This definition purposefully leaves out the presence (or absence) of scalp EEG changes, as clinical seizures may occur without electrographic changes. On the contrary, with the widespread use of continuous EEG for monitoring critically ill patients, other seizure definitions based purely on electrographic criteria—independent of clinical symptoms—have emerged.^10^, ^139^ In this section, we will briefly review the theory behind seizure generators, followed by the review of individual ictal EEG patterns (as recorded on scalp EEG) according to their onset and clinical manifestations, when applicable. Table 5 summarizes the most common ictal EEG patterns.

**TABLE 5:** Summary of ictal EEG patterns.

Generalized onset ictal patterns	Electrographic description	Most commonly associated semiology
Spike‐wave		
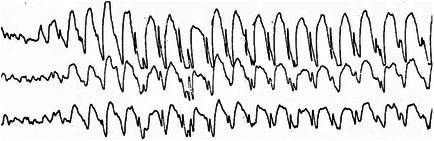	Negative spike followed by a high‐amplitude (200–300 μV) surface negative slow wave	Absence seizure
Polyspike‐wave		
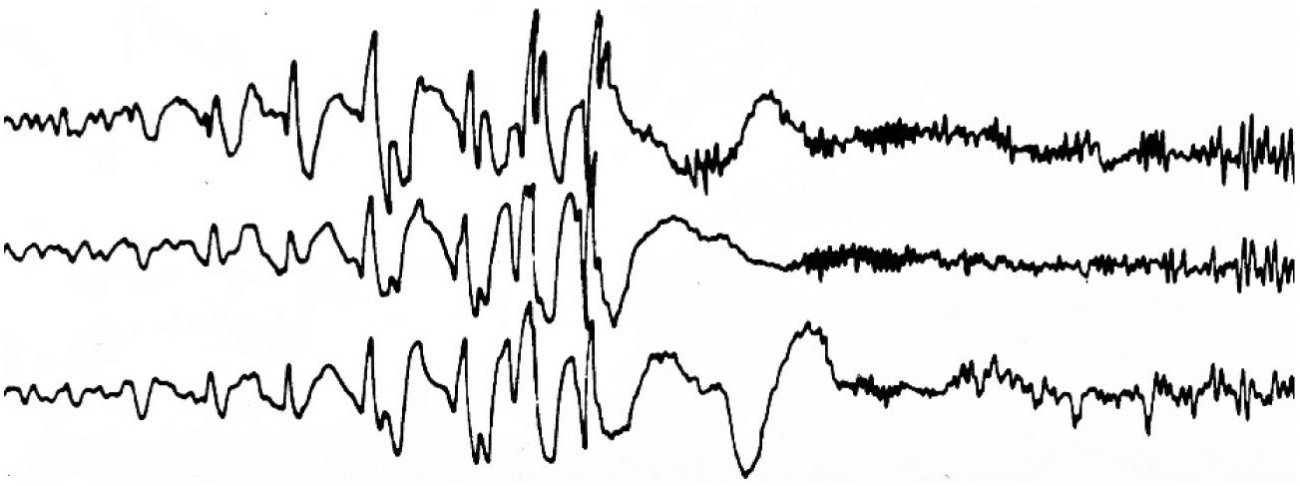	Bilateral polyspikes in succession occurring at 5–20 Hz followed by a slow wave (polyspike–wave complex)	Myoclonic jerk
Generalized paroxysmal fast activity		
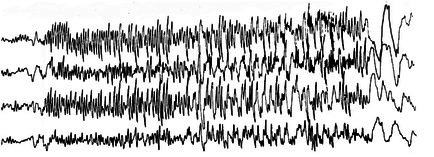	Burst of 15–30 Hz activity that may become obscured by myogenic artifact	Tonic seizure
Generalized fast sharp waves		
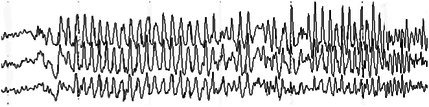	Sharp rhythmic activity at 8–12 Hz followed by diffuse myogenic artifact from tonic muscle contraction	Generalized tonic–clonic seizure
Electrodecrement		
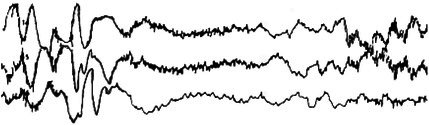	Abrupt amplitude attenuation/suppression usually with superimposed low‐voltage fast activity	Epileptic spasm
**Focal onset ictal patterns** ^a^	**Electrographic description**	**Common associations**
Focal paroxysmal fast activity (focal ictal beta)		
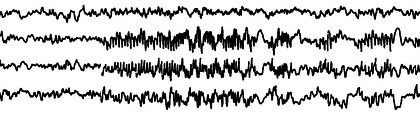	Low‐amplitude focal beta activity	Frontal lobe epilepsy cases with this pattern have better postsurgical outcomes
Repetitive discharges		
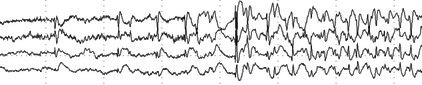	Either (1) appearance of repetitive focal spikes or sharp waves or (2) an increase in interictal spike‐/sharp‐wave frequency with subsequent evolution	
Ebersole and Pacia Pattern 1^c^		
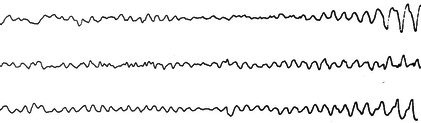	1A: Monomorphic 5–9 Hz rhythmic discharges (if over inferior temporal leads^b^) for a sustained period ≥5 seconds 1B: Monomorphic sharply contoured rhythmic 5‐9 Hz discharges (if at vertex/parasagittal region) 1C: Combination of Type 1A and Type 1B	Mesial temporal lobe onset
Ebersole and Pacia Pattern 2^c^		
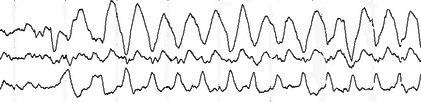	2A: Irregular 2–5 Hz activity lateralized to one hemisphere, that may be 2B) followed by regular theta rhythm (similar to pattern 1) or 2C) preceded by a burst of either irregular or periodc sharp waves	Neocortical temporal lobe onset
Ebersole and Pacia Pattern 3^c^		
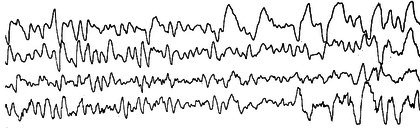	Unlateralized or diffuse irregular changes in background activity	Neocortical temporal lobe onset

^a^ In contrast to generalized ictal patterns, the morphology of focal ictal patterns is not characteristic of a seizure semiology associated with a specific brain region.

^b^ Of a modified 10–20 montage (includes F9/10, T9/10, P9/10).

^c^ For more details, refer to: Ebersole JS, Pacia SV. Epilepsia 1996 Apr;37(4):386–99.

### 4.2 Pathophysiology

#### 4.2.1 Seizure generation

Like most graphoelements observed in scalp EEG, pyramidal neurons with a perpendicular alignment to the cortical surface are the largest contributor to the synchronized activity that will eventually constitute a seizure (Figure 11). At the cellular level, seizures start with a paroxysmal depolarizing shift (PDS), a phenomenon which entails a high‐voltage, long‐lasting depolarization that generates a train of peak action potentials, usually through glutamatergic excitatory neurotransmission, followed by a failure of the surrounding inhibitory milieu.^140^ Spread to distant areas can give rise to manifold clinical manifestations that may at times correlate with the ictal pattern observed on EEG. Ictal patterns function as signatures that aid the clinician in the determination of either a focal onset, generalized onset, combined focal and generalized onset, or unknown onset.

#### 4.2.2 Patterns of seizure onset

Generalized ictal discharges on scalp EEG affect both hemispheres diffusely, which at times may have bilateral regional predominance (e.g., frontocentral). These ictal discharges are frequently associated with changes in frequency and morphology.^141^ What differentiates them from generalized IEDs can be either the presence of clinical signs associated with the pattern or the duration of the pattern.^10^ Focal ictal discharges tend to follow unequivocal dynamic spatiotemporal changes in frequency, morphology, and location, which constitute the concept of *evolution*. To increase specificity, the ACNS recommends using at least two unequivocal, sequential changes in the same direction in at least one of the following domains^142^:

- Frequency: It should change by at least 0.5 Hz in the same direction, two separate times.
- Morphology: It should change to a new pattern two separate times, for example from blunt to sharply contoured and from sharply contoured to spikes.
- Location: It refers to the migration of the ictal discharges into or out of at least two new electrodes (following the standard 10–20 system) for at least three cycles. For example, rhythmic discharges seen at T7, spreading to include F7 for 3 cycles then F7, T7, and P7 for 3 cycles.

Although this framework is useful for academic purposes, the reality of clinical practice is more nuanced. For example, in IGEs a focal ictal scalp EEG pattern can be seen in up to 17% of patients.^143^ On the contrary, patients with focal epilepsy may have a generalized scalp EEG ictal pattern in up to 22% of cases, mostly restricted to extratemporal cases.^144^

### 4.3 Generalized ictal patterns

#### 4.3.1 Spike‐wave

**FIGURE 29:**
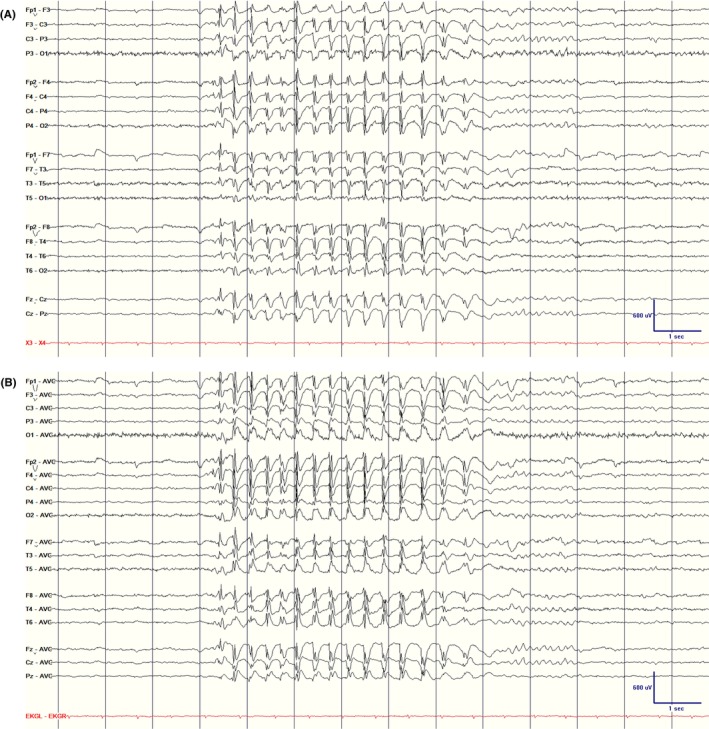
Typical absence seizure in an 8‐year‐old male with behavior arrest. Note that during the first 2 seconds the generalized spike–wave frequency is 3 Hz with subsequent slowing to 2.5 Hz by the end of the seizure. Panel (A): bipolar longitudinal montage; panel (B): common average referential montage. Low‐frequency filter 1 Hz, high‐frequency filter 70 Hz, sensitivity 30 μV/mm.

Introduced by Gibbs, Davis and Lennox in their seminal work titled “The electro‐encephalogram in epilepsy and in conditions of impaired consciousness,” generalized spike‐wave discharges were described as the hallmark ictal pattern of absence seizures.^145^ In the context of generalized epilepsy, the common substrate for the generation of the spike‐wave rhythm is the hijacking of functioning thalamocortical networks, which disrupts consciousness as a result.^146^ For example, typical absence seizures are thought to represent a pathologic substitution of sleep spindles with spike‐wave discharges, where some of the waveforms fuse to form the spike component and some fuse to provide the slow wave.^147^ On EEG, a typical absence seizure is seen as a sequence of a surface negative spike, which may be polyphasic containing ≤3 phases followed by a high‐amplitude (200–300 μV) surface negative slow wave repeating itself at a classic rate of 3 Hz (Figure 29).^148^ Some variation at the beginning of the ictal discharge may be seen with a slight increase in the frequency of the spike‐wave up to 4 Hz with gradual slowing by the end of it and attenuation of the spike component.^148^ There are no significant differences in the duration of absence seizures in CAE vs. JAE.^149^

**FIGURE 30:**
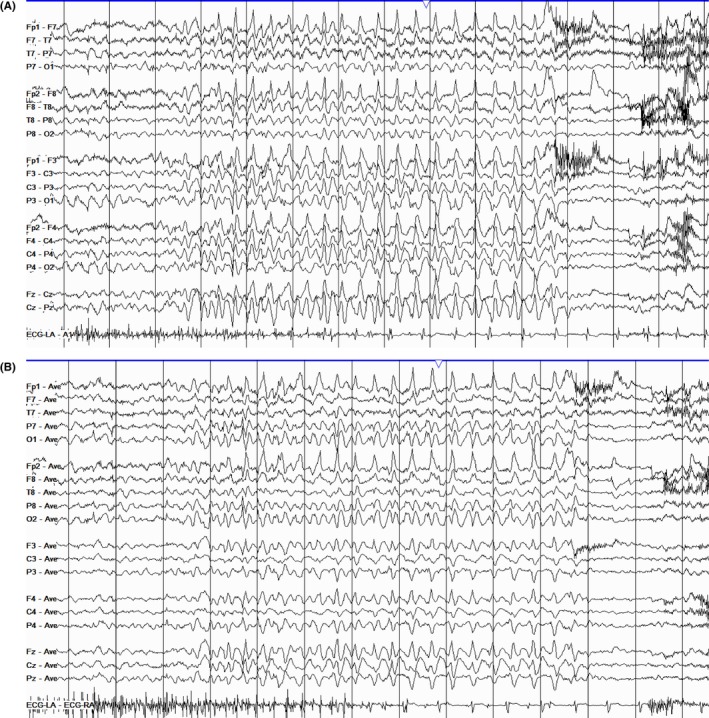
Atypical absence seizure in a 22‐year‐old male with Lennox–Gastaut syndrome. In this case, the generalized discharges are bifrontal predominant and occur at 2.5 Hz. Panel (A): bipolar longitudinal montage; panel (B): common average referential montage. Low‐frequency filter 1 Hz, high‐frequency filter 70 Hz, sensitivity 10 μV/mm for panel A and 15 μV/mm for panel B.

Slow spike‐wave (≤2.5 Hz) patterns represent a variation of the typical absence rhythm discussed above and are seen in DEEs, particularly LGS. Although the substrate for the ictal discharge is the same in the sense that thalamocortical circuitry is hijacked by seizure activity, the circuits themselves seem to have different thalamic relay nuclei with different outputs.^150^ In slow spike‐wave discharges, the key nuclei that reverberate the spike‐wave cycle are the nucleus reuniens of the thalamus and the medial thalamus that directly project to the hippocampus.^151^ Gibbs, Gibbs, and Lennox described the slow spike‐wave pattern as complexes of a blunt spike, followed by a slow hump, repeating itself approximately two times per second.^152^ The spike component is more consistent with a surface negative sharp wave (70–200 msec) with a deep positive deflection followed by a surface negative high‐amplitude slow wave. The ictal discharges tend to have blurred borders rather than the exquisite onset and offset seen in typical absence seizures (Figure 30).^153^

#### 4.3.2 Generalized paroxysmal fast activity

**FIGURE 31:**
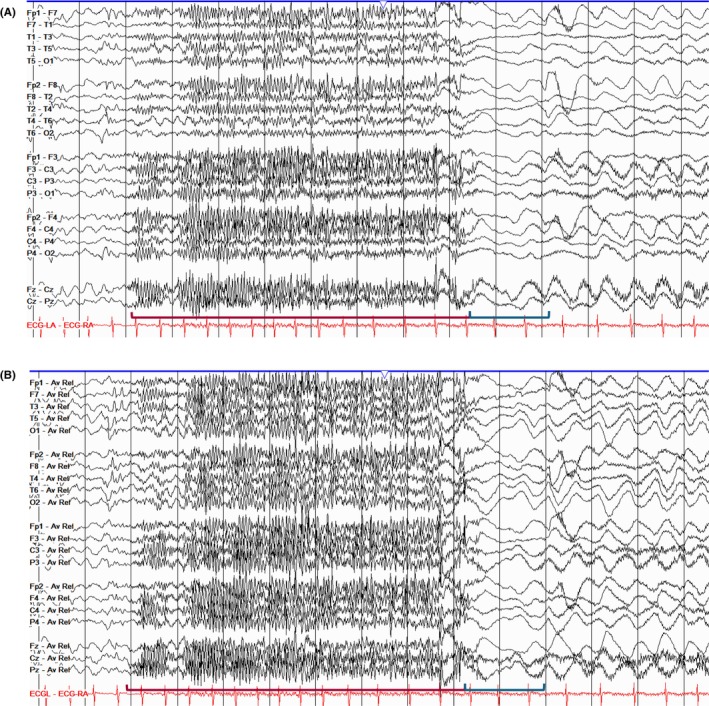
Generalized tonic seizure involving the neck and trunk and subtle elevation movement of the upper extremities in a 22‐year‐old patient with Lennox–Gastaut syndrome. Note the generalized paroxysmal fast activity (red bracket) followed by diffuse attenuation of the background activity (blue bracket) and then generalized slowing. Panel (A): bipolar longitudinal montage; panel (B): common average referential montage. Low‐frequency filter 1 Hz, high‐frequency filter 70 Hz, sensitivity 15 μV/mm.

Like many other findings, the first description of GPFA was published by Gibbs, Gibbs, and Lennox as a fast high‐amplitude discharges that can be seen with or without a clinical seizure (Figure 31).^154^ Initially named “Grand Mal Discharge” due to its association with the initially tonic phase of the tonic–clonic seizure, GPFA is morphologically characterized by a burst of fast activity in the 15–30 Hz range that usually lasts between 1 and 18 s with a mean of 3.1 s.^155^ The clinical correlate of ictal GPFA and *sine qua non* manifestation of LGS is the tonic seizure.^156^, ^157^ However, other clinical manifestations may be seen with GPFA, such as atonic seizures,^158^ versive head turn to either side, urinary incontinency, eye flutter, and respiratory changes.^155^ GPFA, both as ictal and interictal finding, tends to have a predilection for appearing in sleep.^155^ Refer to (Figure 31) for an example of a tonic seizure associated with GPFA.

#### 4.3.3 Generalized electrodecrement

**FIGURE 32:**
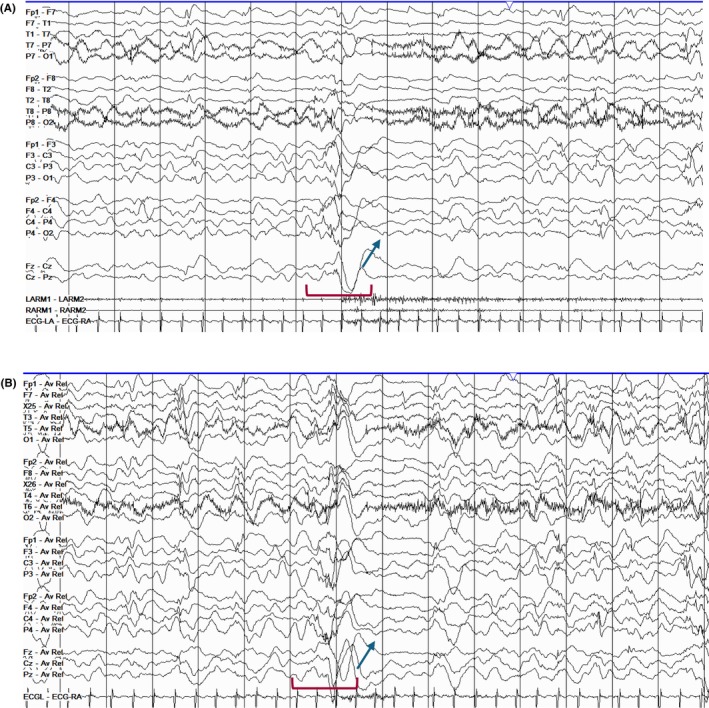
Epileptic spasm in a 3‐month‐old infant. Note the diffuse, one‐second‐long attenuation of the background (arrow) after a sharp‐wave complex (red bracket). This was associated with sudden shoulder elevation and neck flexion spasm. Panel (A): bipolar longitudinal montage; panel (B): common average referential montage. Low‐frequency filter 1.6 Hz, high‐frequency filter 70 Hz, sensitivity 30 μV/mm.

This ictal EEG finding represents a period of amplitude attenuation usually with superimposed low‐voltage fast activity.^43^ Generalized electrodecrement is the typical pattern observed in infantile spasms, and while the attenuation phase tends to be relatively preserved across seizures, there is significant variability in what surrounds it.^159^ The most common pattern associated with epileptic spasms is that of a slow wave followed by diffuse attenuation (Figure 32), which can be seen in about a third of cases. However, electrodecrement can also be associated with slow spike‐wave and/or fast activity before or after the attenuation.^159^ Additionally, electrodecrement can have a generalized or focal distribution and can also be seen in some focal onset epilepsies. In studies where simultaneous intracranial EEG and scalp EEG electrodes are recorded, scalp electrodecrement correlates with low‐voltage fast activity on the intracranial recording. Nodding syndrome has also been associated with electrodecrement as an ictal pattern that starts with a diffuse, broad slow wave with subsequent attenuation and superimposition of gamma activity that clinically translates into vertical head nods.^160^ Head nodding syndrome is a childhood‐onset epilepsy syndrome characterized by multiple attacks of vertical head drops with or without tonic–clonic seizures, developmental delay, and stunned growth predominantly seen in eastern Africa.^161^ Electroclinically, nodding syndrome is thought to be related to late‐onset infantile spasms.^160^

#### 4.3.4 Polyspikes

**FIGURE 33:**
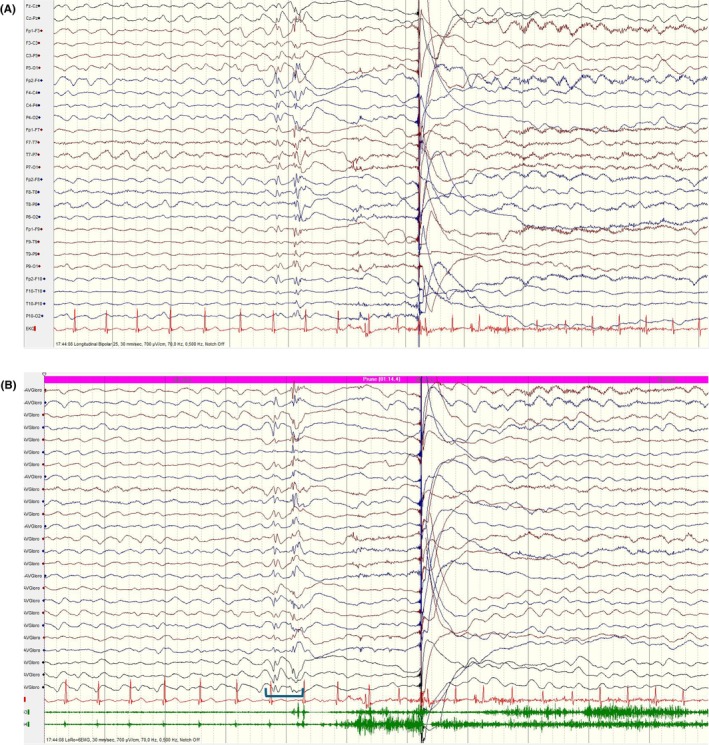
Myoclonic‐atonic seizure in an 8‐month‐old infant. Note the generalized polyspike–wave burst (bracket) leading into a myoclonic jerk noted in the EMG lead followed by electrodecrement with dropout on the EMG lead. Panel (A): bipolar longitudinal montage; panel (B): common average referential montage. Low‐frequency filter .5 Hz, high‐frequency filter 70 Hz, sensitivity 7 μV/mm.

Probably, the first ictal description of polyspike discharges as an ictal phenomenon was that of Dieter Janz in 1959 defined as “Impulsiv‐Petit mal.”^162^ The typical discharge is made of multiple bilateral, frontal predominant spikes in succession occurring at 5–20 Hz followed by a slow wave, known as the polyspike–wave complex.^163^ The classic manifestation of polyspike discharges is a quick startle‐like jerk, predominantly of arms and shoulders with preserved consciousness, which, in the case of JME, tends to occur after waking up.^163^ However, myoclonus associated with polyspikes is not exclusive to JME and may be seen in other conditions including developmental epileptic encephalopathies (e.g., epilepsy with myoclonic‐atonic seizures (EMAtS)) (Figure 33),^52^ anoxic brain injury,^164^ toxic ingestions,^165^ progressive myoclonic epilepsies,^166^ among others.^167^

#### 4.3.5 Generalized fast sharp waves

**FIGURE 34:**
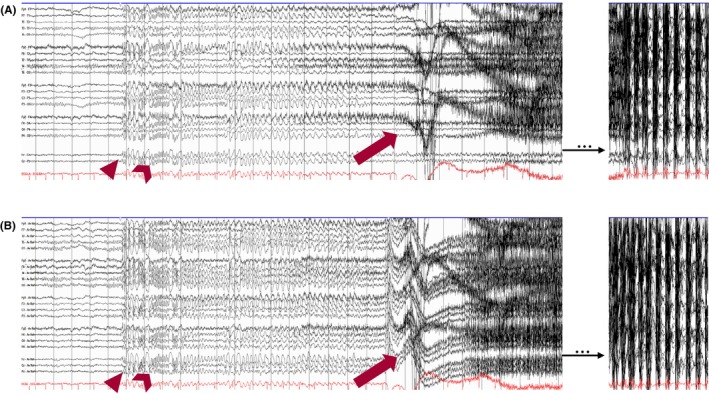
Generalized tonic–clonic seizure in a 42‐year‐old patient. At the onset, note the generalized polyspike–wave discharges at 4–5 Hz (arrowhead) leading into frontally predominant alpha frequency rhythmic sharp waves (chevron) followed by rhythmic delta‐theta activity that evolves in frequency before it is superimposed by myogenic artifact from tonic phase (arrow). Later, during the clonic phase, myogenic artifact becomes apparent. Panel (A): modified longitudinal bipolar montage with anterior temporal electrodes; panel (B): common average referential montage. Low‐frequency filter 1 Hz, high‐frequency filter 70 Hz, sensitivity 10 μV/mm.

Primary tonic–clonic seizures are rarely captured in scalp video‐EEG recordings as they tend to be associated with other seizure semiologies that occur more frequently and are more specific to a determined epilepsy syndrome. For example, if a teenager comes to clinic after a tonic–clonic seizure and the a video‐EEG records a polyspike‐wave associated with a myoclonic jerk with interictal frontally predominant 4.5 Hz spike/polyspike–wave discharges, a diagnosis of JME can be made without the need to capture a generalized tonic–clonic seizure.^50^ In the rare instances when generalized tonic–clonic seizures are captured, these tend to be preceded by either a run of spike–wave discharges^168^ or a by rhythmic repetitive sharp waves, such as in the historical description of the *Grand Mal* discharge by Gibbs, Gibbs, and Lennox (Figure 34).^145^ The repetitive sharp waves are generalized or frontally predominant and occur at a frequency of 5–18 Hz. Any ictal discharge will most likely become obscured by myogenic artifact soon after onset.

### 4.4 Focal ictal patterns

#### 4.4.1 Ictal patterns in temporal lobe epilepsy

Temporal lobe epilepsy, being the most common focal epilepsy in the adult patient population, is the focal epilepsy where scalp EEG is the most useful for lateralization and localization of the epileptogenic zone given distinct patterns that can be seen depending on the origin of the ictal rhythm with respect to the temporal lobe itself. For example, using temporal or sphenoidal electrodes, Risinger et al. accurately determined the laterality of a temporal seizure onset in 82% of the time if the ictal discharges reached rhythmic ≥5 Hz within the first 30 seconds of onset^169^ Furthermore, the best description of ictal rhythms determining the localization of temporal lobe seizure is the one by Ebersole and Pacia in 1995, where three basic ictal rhythms are proposed.^170^

**FIGURE 35:**
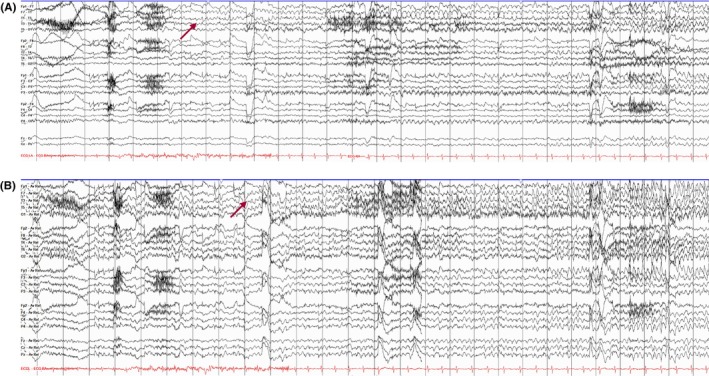
Left temporal seizure with type 1 pattern according to Ebersole and Pacia. Note the rhythmic sharp theta activity starting at T3 (arrow) with subsequent spread to the rest of the temporal chain. Panel (A): modified longitudinal bipolar montage that includes anterior temporal electrodes; panel (B): common average referential montage. Low‐frequency filter 1 Hz, high‐frequency filter 70 Hz, sensitivity 7 μV/mm for panel A, and 10 μV/mm for panel B.

**FIGURE 36:**
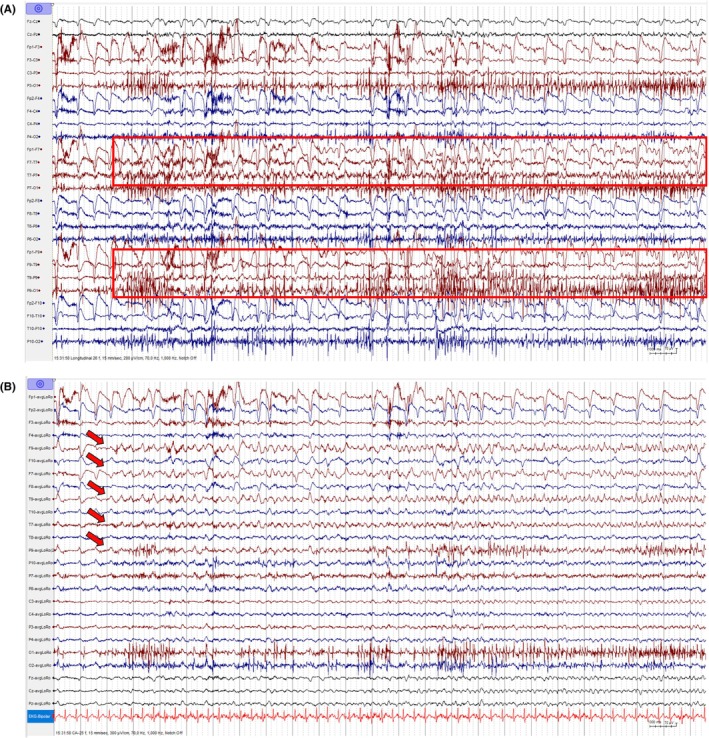
Left temporal seizure starting with rhythmic delta sharply contoured activity (rectangles on panel A, arrows on panel B) with evolution in frequency, morphology, and location consistent with Ebersole and Pacia type 2 pattern. This pattern is less specific for mesial temporal onset and can be associated with temporal neocortical onset. Panel (A): bipolar longitudinal montage; panel (B): common average referential montage. Low‐frequency filter 1 Hz, high‐frequency filter 70 Hz, sensitivity 15 μV/mm.

**FIGURE 37:**
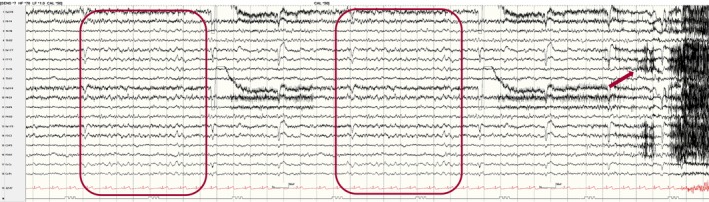
Focal seizure of unclear onset on EEG (Ebersole and Pacia type 3 pattern). The semiology was consistent with a focal seizure with impaired awareness associated with inability to talk. After the patient underwent stereo‐EEG, a left basal temporal focus was noted, and the patient underwent a temporal lobectomy with postoperative seizure freedom. Note the presence of bilateral frontotemporal slowing (red rectangles) that fluctuates and becomes obscured by myogenic artifact (arrow). Bipolar longitudinal montage, Low‐frequency filter 1 Hz, high‐frequency filter 70 Hz, sensitivity 7 μV/mm.

1. Type 1 pattern is defined by either: monomorphic 5–9 Hz rhythmic discharges over the inferior temporal leads of a modified 10–20 montage (F9/10, T9/10, P9/10) for a sustained period ≥5 s (Type 1A); or rhythmic 5 Hz frontocentral monomorphic sharply contoured discharges (Type 1B; or a combination of Type 1A and Type 1B—Type 1C).^170^ This pattern is highly associated with a hippocampal (mesial) temporal lobe onset (Figure 35).^170^
2. Type 2 pattern is defined by irregular 2–5 Hz diffuse hemispheric discharges, at times maximal in the temporal electrodes with multiple changes in morphology every few seconds occurring in isolation (Type 2A), or that may be followed by rhythmic 5–9 Hz temporal/subtemporal maximal activity resembling that of Type 1A (Type 2B); or that may be preceded by lateralized periodic sharp waves or a burst of irregular sharp waves lasting 1–2 s (Type 2C). This pattern is associated with a neocortical temporal lobe onset (Figure 36).
3. Type 3 pattern is defined as seizure onset where there is diffuse, often irregular, background changes and slowing at the time of clinical onset. This pattern was associated with neocortical temporal lobe onset (Figure 37).

#### 4.4.2 Other focal ictal patterns

**FIGURE 38:**
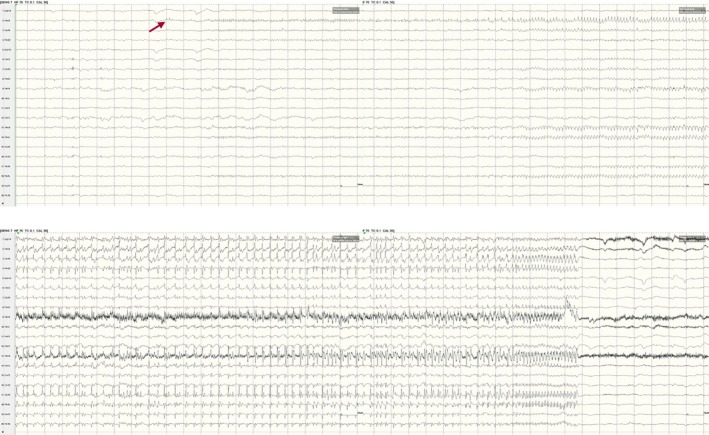
Right frontal electrographic seizure on a bipolar montage. Note the sharp rhythmic activity maximal at F4 at the beginning of the seizure (arrow) subsequently evolving in frequency, morphology, and location (into diffuse rhythmic spiking) with abrupt offset (trace below). Bipolar longitudinal montage. Low‐frequency filter 1 Hz, high‐frequency filter 70 Hz, sensitivity 7 μV/mm.

Studies evaluating the reliability of scalp EEG to localize and lateralize seizures have shown a significantly less robust agreement when it comes to extratemporal seizures vs. temporal onset seizures.^171^ However, once ictal discharges are clear, they will very likely follow one of the following ictal patterns: (a) repetitive spiking, (b) evolving rhythmic discharges (Figure 38), (c) focal paroxysmal fast activity (also known as focal ictal beta),^172^, ^173^ or (d) focal electrodecrement.^18^ Neither of these patterns is specific to a lobar region, and we can expect to see them with both temporal and extratemporal epilepsy.

## 5 PROVOCATION MANEUVERS

Provocative maneuvers are integral to standard EEG recording procedures. These maneuvers are designed to elicit specific responses that can aid in the diagnosis of epilepsy.

### 5.1 Hyperventilation

**FIGURE 39:**
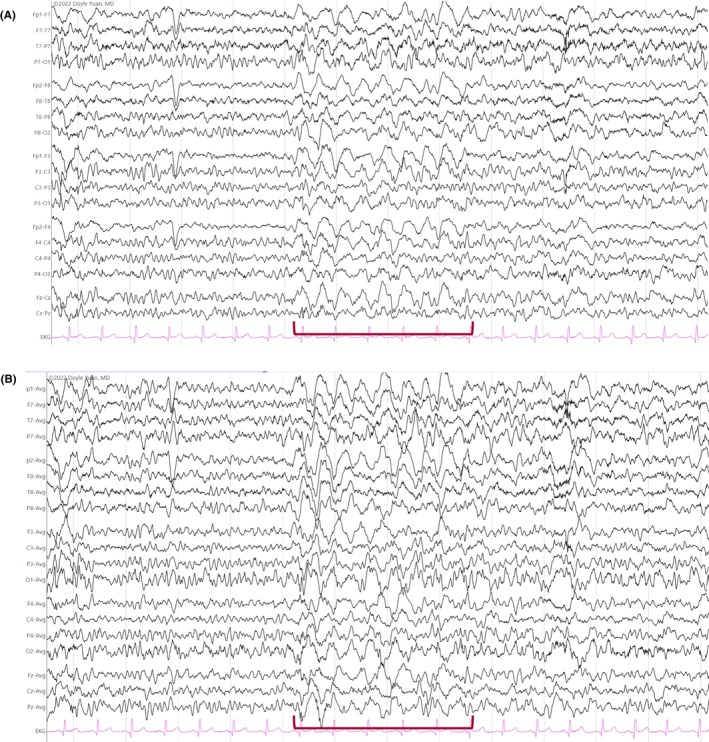
Hyperventilation‐induced generalized delta slowing (red bracket) as seen in a 25‐year‐old patient with normal EEG after 1 minute of hyperventilation with good effort. Panel (A): bipolar longitudinal montage; panel (B): common average referential montage. Low‐frequency filter 1 Hz, high‐frequency filter 70 Hz, sensitivity 7 μV/mm.

Hyperventilation is a commonly used provocative maneuver in routine EEG tests. Hyperventilation may induce epileptiform abnormalities in individuals with epilepsy syndromes, such as CAE and other generalized epilepsies.^83^ During hyperventilation, the patient is asked to breathe deeply and rapidly for 3–5 min, which typically induces diffuse, bilateral high‐amplitude theta and delta slowing on the EEG.^174^ The EEG recording should be continued for at least 1–2 min after stopping hyperventilation as abnormalities may appear during the immediate post‐hyperventilation period.^175^ Hyperventilation‐induced slowing is most often observed in children, especially those aged 8 to 12 years, but can also be seen in young adults (Figure 39) The underlying mechanism is believed to involve hypocapnia, which may either activate thalamocortical projecting systems or decrease activity in the mesencephalic reticular formation, leading to subsequent slowing as seen on EEG.^3^ Hyperventilation‐induced slowing is generally considered normal unless it (a) presents as focal or unilateral, (b) is significantly asymmetric, or (c) is accompanied by epileptiform discharges.^3^ Additionally, fasting and possibly low blood glucose levels (<80 mg/dL) enhance the likelihood of this response even in adults.^176^, ^177^ The hyperventilation maneuver is contraindicated in conditions like recent symptomatic cerebrovascular diseases, including intracranial hemorrhage or inflammatory vasculitis, such as Moya‐Moya disease, sickle‐cell disease, or cardiopulmonary disease.^174^

### 5.2 Photic stimulation

**FIGURE 40:**
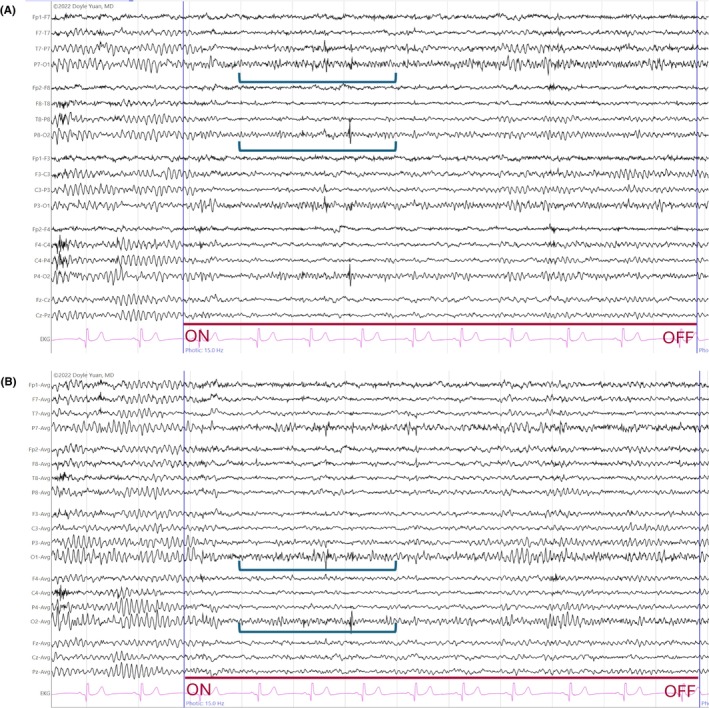
Photic driving (blue bracket), a normal EEG response seen with photic stimulation at a frequency of 15 Hz (red line). Panel (A): bipolar longitudinal montage; panel (B): common average referential montage. Low‐frequency filter 1 Hz, high‐frequency filter 70 Hz, sensitivity 7 μV/mm.

Photic stimulation is another common activation maneuver used in routine EEG. During this procedure, a strobe light is positioned in front of the patient's head, and flashing lights at various frequencies are shown in sequential bursts with the patient's eyes open and closed. For optimal results, photic stimulation should be conducted in a room with dim lighting, with the lamp positioned at least 30 cm from the patient's face.^175^ Photic driving, a normal variant, is an evoked cerebral response defined as rhythmic activity seen in occipital regions that is time‐locked with intermittent photic stimulation (IPS), with a small time lag. The driving response is of a frequency that is identical or harmonically related to the IPS frequency and is considered the most common response to IPS (Figure 40). The photic driving response typically occurs at frequencies between 5 and 30 Hz and typically maximal around the patient's PDR frequency.^3^ A photomyogenic response, which includes repetitive muscle artifacts from frontalis muscle contraction and eye blinking in response to the flashing lights, is best seen in the anterior leads on EEG and can also be seen in response to IPS. This is also known as photomyoclonic response, photo‐oculoclonic response, and orbitofrontal photomyoclonus and is considered a normal response.^3^ Photomyogenic response has been most frequently observed in patients experiencing early alcohol withdrawal or increased anxiety.^178^

**FIGURE 41:**
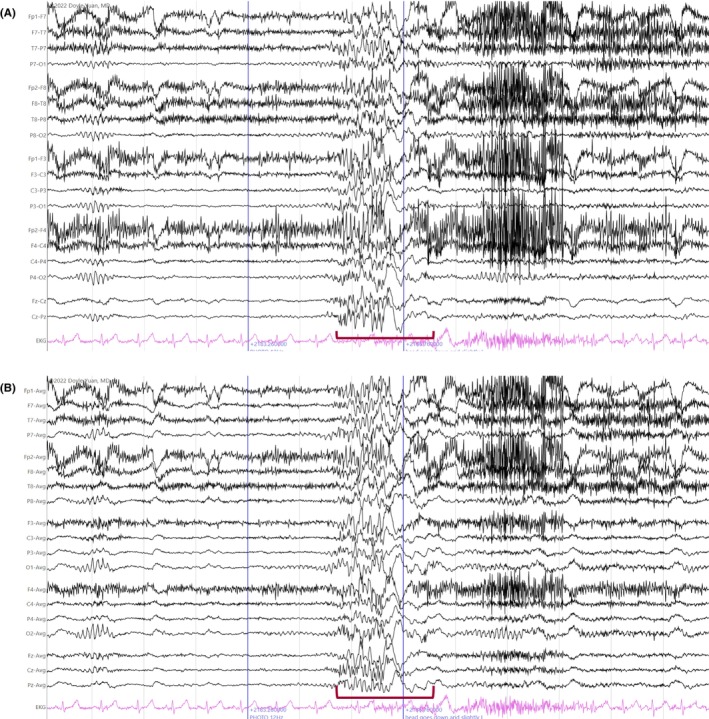
Photoparoxysmal response in a 16‐year‐old patient characterized by generalized polyspike–wave discharges on EEG (red bracket) seen at photic stimulation at a frequency of 12 Hz. Panel (A): bipolar longitudinal montage; panel (B): common average referential montage. Low‐frequency filter 1 Hz, high‐frequency filter 70 Hz, sensitivity 15 μV/mm.

Photoparoxysmal response is a pathological response to photic stimulation characterized by the presence of epileptiform discharges and/or seizures not linked to photic stimulation frequency during photic stimulation that may or may not persist after the stimulation ends (Figure 41). This is usually seen in GGEs. Photosensitivity can also be seen in photosensitive occipital lobe epilepsy, a type of focal reflex epilepsy, and EEM.^179^ This response is highly dependent on the luminance of the stimulating light, the frequency of photic flicker (maximal from 15 to 18 Hz), and relates to the wavelength of the light.^180^

### 5.3 Sleep deprivation

Including sleep in EEG recordings enhances diagnostic yield, as certain EEG abnormalities are more likely to be detected during sleep and awake‐sleep transitions. Sleep deprivation prior to EEG recording also increases the detection of abnormalities.^174^ Although sleep recordings are helpful for detecting epileptiform discharges, they should be used in addition to, rather than as a substitute for, wakeful recordings. Studies have shown that EEGs performed after sleep deprivation had an improved yield of detection of epileptiform abnormalities, with up to 35% of adults and children showing abnormal findings post‐deprivation when initial EEGs were normal.^70^

### 5.4 Other provocation maneuvers

Evaluating for fixation‐off sensitivity and eye‐closure sensitivity is particularly important in patients with occipital lobe epilepsy and generalized epilepsies, such as EEM, as epileptiform discharges may be triggered in predisposed individuals when the eyes are closed or when central vision and fixation are suppressed.^181^, ^182^ These assessments can be performed using techniques like eyelid closure, total darkness, or the utilization of Frenzel lenses.^3^, ^179^

Specific provocation techniques tailored to individual triggers can be beneficial to patients with reflex seizures. An example of this is reading epilepsy, a condition that primarily affects individuals between the ages of 12 and 20 years. It is characterized by seizures that are provoked by reading or language‐related activities and often manifest as reflex myoclonus. Similarly, myoclonic epilepsy in infancy, affecting children aged 3–36 months, can be triggered by unexpected auditory or tactile stimuli.^179^

Provocative techniques have been used for eliciting and diagnosing psychogenic non‐epileptic seizures (PNES), especially when spontaneous paroxysmal events do not occur during video‐EEG monitoring. Methods such as placement of an alcohol‐infused pad on the neck, placement of a vibrating tuning fork on the head, photic stimulation, hyperventilation, and verbal suggestion utilize the susceptibility trait of somatoform diseases to provoke episodes. The use of such techniques is debated due to associated ethical concerns given their potentially deceitful nature, which may be addressed by clearly explaining the technique to the patient as a nocebo. Techniques that involve even negligible excess risk, such as injection of normal saline, should not be utilized.^183^

## 6 FUTURE PERSPECTIVES

### 6.1 Other interictal markers of epileptogenicity

**FIGURE 42:**
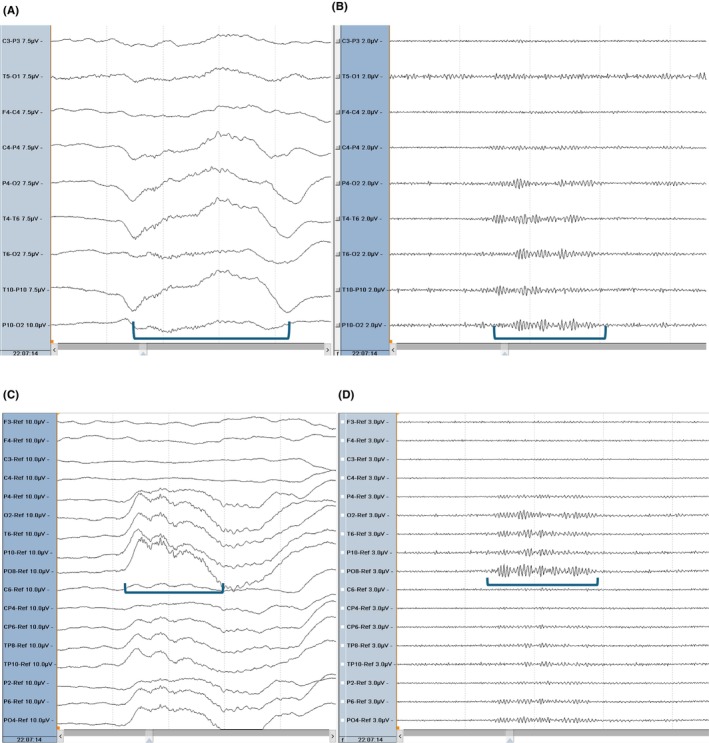
Example of scalp high‐frequency oscillations (HFOs) recorded from a 27‐year‐old left‐handed patient with non‐lesional right posterior quadrant neocortical epilepsy. Panel (A) is an excerpt of a high density (10–10 system) EEG with a right posterior quadrant (on average montage, maximal over T6 and P10 > O2) sharp wave (blue bracket) with superimposed high‐frequency oscillations >80 Hz, panel (B) shows the same segment as panel A after using a finite impulse response (FIR) 80 Hz low‐pass filter allowing a clear identification of the HFOs (blue bracket). Panels (C) and (D) show the same sharp wave and HFOs in a referential common average montage in unfiltered and filtered version.

It has been close to 100 years since Hans Berger published his first report of the human electroencephalogram.^184^ Since then, the description of new EEG biomarkers to aid in the care of people living with epilepsy has been relatively limited, despite the widespread availability of EEG. Most of the “classic” interictal abnormalities mentioned in this seminar take place between the 0.5 and 70 Hz window, which is the IFCN and ILAE recommended visualization filtering.^185^ High‐frequency oscillations (HFOs), defined as spontaneous EEG activity between the 80 and 500 Hz frequency band,^186^ have been utilized in the evaluation of epilepsy surgery patients mainly through intracranial electrodes as a means to delineate the seizure onset zone with even higher specificity than interictal spikes.^187^ During the past two decades, HFOs have also been observed in scalp EEG in both children^188^ and adults (Figure 42).^189^ This has opened the possibility of expanding the non‐invasive assessment of the epileptogenic zone as well as monitoring the clinical status of patients living with epilepsy.^190^ However, given the multiple technical limitations of recording HFOs on scalp EEG, such as low signal to noise ratio, need for automated quantification, and lack of standardization in their role within the epilepsy surgery workup, the use of scalp HFOs remains investigational and not ready for clinical use.^190^

### 6.2 Automatization of EEG interpretation with artificial intelligence

**FIGURE 43:**
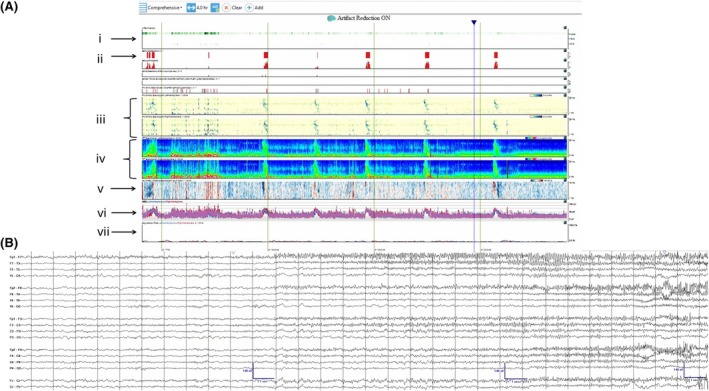
Multimodal quantitative EEG analyzing 4 hours of EEG per bar (panel A). The different channels are: (i) myogenic artifact band, (ii) automatic seizure detection and seizure probability, (iii) rhythmicity spectrogram, (iv) fast Fourier transform (FFT) spectrogram, (v) relative asymmetry spectrogram, (vi) automatic EEG (aEEG) band comparing hemispheres, and (vii) suppression ratio. Note how there is a red square in the seizure detection (ii) that is synchronous with a band narrowing in aEEG that initially shows a left sided prominence (seen as a dark blue band in the asymmetry spectrogram; v) giving the characteristic flame pattern in the FFT (iv). Panel B shows the corresponding seizure on raw EEG, which starts as focal fast activity starting in the left frontal region with fast bifrontal spread.

With the increase in critical care EEG over the years, the need for interpretation of substantial amounts of continuous EEG data has become critical for inpatient clinical practice. To ease the burden of scanning every page/screen of recording, quantitative EEG data processing using Fourier transforms in addition to automatic spike and seizure detection has become widely available and utilized in clinical practice (Figure 43).^191^ However, even after the data have been distilled and carefully packaged into a more digestible display, the neurophysiologist or epileptologist still has to vet and interpret the data as well as generate a report. As an attempt to automatize EEG interpretation, Shibasaki et al. created an algorithm that combines band power assessment for background inspection, automatic spike detection, and artifact elimination to analyze routine EEG recordings.^192^ This system has the limitation that it only analyzes awake EEG segments and that it is a fixed model that requires ongoing human modification of the neural network model if a specific interpretation aspect needs to be modified.^192^ With the advent of artificial intelligence, multiple applications in clinical neurology have been devised for its use. Within the realm of electroencephalography, the development of the Standardized Computer‐based Organized Reporting of EEG–Artificial Intelligence (SCORE‐AI) model represents the first validated convolutional neural network model that is able to automatically analyze routine EEG recordings (~30 min duration) and provide an automatic report that classifies the recording as: (1) focal epileptiform, (2) generalized epileptiform, (3) focal slow, (4) generalized slow, or (5) normal, without the need of human intervention, and with results that are comparable to human experts in EEG.^193^ In the realm of ICU EEG monitoring, SPaRCNet is a neural network algorithm that distinguishes seizures and periodic patterns as well as robustly identifies the ictal–interictal continuum (IIC).^194^ Artificial intelligence could potentially bridge the gap in EEG access in underserved areas and help lower the workload for busy practices by stratifying recordings that require a human second look.^195^ SCORE‐AI was developed using over thirty thousand annotated EEGs and validated using close to ten thousand recordings that were not part of the model training; however, the system is still limited by the fact that only routine EEGs can be analyzed and not the more complex long‐term video‐EEG monitoring or EMU inpatient recordings.^193^ In the future, ultralong‐term EEG recordings with fewer channels and more detailed reports delineating specific lobar locations may be of high yield.^196^

## 7 CONCLUSIONS

In this Seminar of Epileptology, we have provided the reader with the basics regarding normal EEG findings in wakefulness and sleep, interictal non‐epileptiform and epileptiform abnormalities, as well as the most common seizure patterns on scalp EEG. This review does not intend to be a comprehensive resource; rather, it provides a quick reference to the most encountered normal findings seen in wakefulness and sleep, as well as common non‐epileptiform and epileptiform abnormalities in scalp EEG recordings. We suggest readers seek further information from specialized references when needed.^18^, ^197^, ^198^ The accurate interpretation of scalp EEG recordings is a stepping stone in ensuring optimal epilepsy care. We hope this seminar will facilitate this important skill.

## CONFLICT OF INTEREST STATEMENT

None of the authors has any conflict of interest to disclose that is pertinent to the contents of the manuscript. No funding was allocated toward the conceptualization or production of the present work. RK and FAN are Associate Editors of Epileptic Disorders. BF is Deputy Editor of Epileptic Disorders. SB is Editor‐in‐Chief of Epileptic Disorders.

**Test yourself**

1. A 9‐year‐old child undergoes a routine EEG. An arciform waveform in the alpha frequency range is seen over the central regions, shifting from side to side and blocked when the child moves their contralateral hand. What is the most likely rhythm identified?
  - Posterior dominant rhythm
  - Mu rhythm
  - Sleep spindle
  - Beta rhythm
2. Which of the following EEG features is most characteristic of REM sleep?
  - Vertex sharp waves
  - Sleep spindles
  - Sawtooth waves with 2–6 Hz frequency and serrated appearance
  - Generalized spike‐wave discharges
3. A 14‐year‐old with juvenile myoclonic epilepsy undergoes an EEG. Which of the following findings would most strongly support the diagnosis?
  - Consistently unilateral focal spikes
  - Diffuse background slowing without sedation
  - 3–5.5 Hz generalized spike‐wave or polyspike‐wave complexes
  - Generalized slow spike‐wave <2.5Hz not at the end of a burst
4. A 6‐month‐old infant's EEG shows sleep spindles that are asynchronous and fluctuate in amplitude. What is the most appropriate interpretation?
  - This pattern is abnormal and suggests focal pathology
  - The morphology is consistent with normal spindle development
  - Sleep spindles should be symmetric and synchronous by this age
  - Spindles are not expected until after 12 months of age
5. A 5‐year‐old presents with daily drop attacks and developmental delay. EEG shows generalized slow spike‐and‐wave complexes <2.5Hz and paroxysmal fast activity in sleep. What is the most likely diagnosis?
  - Epilepsy with myoclonic absences
  - Lennox–Gastaut syndrome
  - Childhood absence epilepsy
  - Epilepsy with myoclonic‐atonic seizures
6. Which of the following EEG findings is most characteristic of hypsarrhythmia?
  - Generalized 3‐Hz spike‐and‐wave discharges
  - High‐amplitude slow waves with multifocal spikes, disorganized background
  - Generalized paroxysmal fast activity during REM sleep
  - Polymorphic theta with lateral rectus spikes
7. A 4‐year‐old with cognitive regression has EEG during sleep showing slow spike‐and‐wave at 1.5–2 Hz markedly activated in non‐REM sleep. What is the most likely diagnosis?
  - Developmental and epileptic encephalopathy with spike‐and‐wave activation in sleep (SWAS)
  - Epilepsy with myoclonic‐atonic seizures
  - Childhood absence epilepsy
  - Febrile infection‐related epilepsy syndrome
8. Which of the following best defines an epileptiform discharge based on the 5 IFCN criteria?
  - Brief, sharply contoured waveform without background disruption
  - Pointed morphology, asymmetrical waveform, after‐going slow wave, background disruption, and a field
  - Sharply contoured symmetrical waveform without field
  - Repetitive alpha waves during drowsiness
9. An EEG pattern is evolving in frequency from 5 Hz to 3 Hz and then 1 Hz and migrating from T7 to F7 to P7 over 15 seconds. What term best describes this pattern?
  - Generalized epileptiform discharge
  - Multifocal sharp wave pattern
  - Ictal pattern with spatiotemporal evolution
  - Hypsarrhythmia
10. Which EEG feature best defines a periodic pattern such as lateralized periodic discharges (LPDs)?
  - Irregular discharges lacking a fixed interval
  - Brief rhythmic discharges lasting under 3 cycles
  - Repetition of a waveform at regular intervals continuing for at least 6 cycles
  - Background rhythm disrupted by isolated spikes
11. Which of the following is a normal EEG finding during drowsiness in adults?
  - Persistent generalized delta slowing
  - Frontal spike‐wave discharges
  - Roving eye movements
  - Sleep spindles
12. What EEG finding is considered abnormal during wakefulness?
  - Lambda waves
  - Generalized polymorphic delta slowing
  - Mu rhythm
  - Posterior dominant rhythm

Answers may be found in the *supporting information*.

## Supporting information


Data S1.

## Data Availability

Data sharing is not applicable to this article as no new data were created or analyzed in this study.
