# Supplementary material for: Seminar in Epileptology: Normal awake and sleep patterns, interictal abnormalities, and ictal patterns on scalp EEG
Source: Epileptic Disord. 2025 Aug 9;27(5):803–66. doi: 10.1002/epd2.70071 (PMC12574496; doi:10.1002/epd2.70071)
Supplement: Supplementary file 1 — Data S1. [file EPD2-27-803-s001.docx]

**Test yourself**

**Answers:**

1. B. **Mu rhythm**

2. C. **Sawtooth waves with 2–6 Hz frequency and serrated appearance**

3. C. **3–5.5 Hz generalized spike-wave or polyspike-wave complexes**

4. B. **The morphology is consistent with normal spindle development**

5. B. **Lennox–Gastaut syndrome**

6. B. **High-amplitude slow waves with multifocal spikes, disorganized background**

7. A. **Developmental and epileptic encephalopathy with spike-and-wave activation in sleep (SWAS)**

8. B. **Pointed morphology, asymmetrical waveform, after-going slow wave, background disruption, and a field**

9. C. **Focal ictal pattern with spatiotemporal evolution**

10. C. **Repetition of a waveform at regular intervals continuing for at least 6 cycles**

11. C. **Roving eye movements**

12. B. **Generalized polymorphic delta slowing**
